# On the mathematical structure and numerical solution of discrete–continuous optimization problems in DDCM

**DOI:** 10.1007/s00033-025-02536-4

**Published:** 2025-08-06

**Authors:** Cristian G. Gebhardt, Senta Lange, Marc C. Steinbach

**Affiliations:** 1 Geophysical Institute and Bergen Offshore Wind Centre (BOW), University of Bergen, Allégaten 70, 5007 Bergen, Norway; 2 Institute of Applied Mathematics, Leibniz Universität Hannover, Welfengarten 1, 30167 Hannover, Germany

**Keywords:** Data-driven elasticity, Discrete–continuous quadratic optimization, Structural analysis, Alternating direction method, 74B20, 74S05, 90C11, 90C20, 90C59

## Abstract

In this work, we investigate data-driven elasticity problems defined on a closed interval of the real line that are spatially discretized by means of the finite element method. This one-dimensional setting allows us to gain a deeper understanding of the underlying *discrete–continuous quadratic optimization problems*. We provide an in-depth analysis of their structural properties and prove their global solvability. Based on this analysis, we propose a new structure-specific initialization for a solution strategy relying on an *alternating direction method*, and we prove that it is globally optimal in certain symmetric cases. Finally and to support our formal mathematical analysis, we also provide a series of examples that show the benefits of this kind of approach and briefly illustrate the challenges when dealing with real experimental data.

## Introduction

*Data-driven computational mechanics*, or DDCM in brief, has emerged as the latest paradigm in computational continuum mechanics. In DDCM, the strain and stress fields over a physical body are then to be found as the closest to some field extension of a given finite set of empirically determined strain–stress pairs. Mathematically speaking, such a problem can be formulated as a non-convex topological optimization problem, in which both the data extensions defined over the continuum and the displacement, strain, and stress fields are to be sought for simultaneously so that the equilibrium and compatibility conditions are satisfied in some weak or strong sense. This optimization problem is in general extremely difficult to analyze and to solve. However, by adopting a finite element discretization and assuming that every domain decomposition on which the extensions of the data are defined can be expressed in terms of the triangulation’s elements, the problem simplifies enormously and becomes analyzable and computationally tractable. To place the ideas of the present work into a suitable context, we describe next some of the most important contributions in the field.

Although DDCM is still in its infancy, it has been around for some time. The first contribution we are going to refer to is the one proposed by Kirchdoerfer and Ortiz [13]. That work introduces a formulation for multi-bar structures relying on the data in itself and two primal variables and a single dual one. This results in a convex quadratic optimization problem that involves discrete optimization variables in addition, i.e., a *discrete–continuous quadratic optimization problem* (DCQP). These authors also propose a solution strategy comprising two steps: *i*) a random initialization, followed by *ii*) an (apparently reinvented) *alternating direction method* (ADM). In [14], the same authors extend that formulation by introducing an annealing schedule resulting in a solution strategy that improves robustness in the presence of material data containing outliers. Finally, in [15], these authors formulate a new variant of DDCM, but this time in the dynamic setting assuming that a constant mass matrix is given. Even when related to the previous work in the static setting, the time-dependent problem is slightly different since it resembles a sort of “hyperbolic” problem with a preferential initialization (by the solution of the previous time step) which is by far better than randomly initialized schemes. In contrast with this, Kanno [11] investigates the same DDCM problem by solving the underlying DCQP to global optimality using a *mixed-integer QP* (MIQP) reformulation and the commercial solver CPLEX. Moreover, in [12] Kanno solves the DDCM problem by means of an ADM combined with local kernel reconstruction of the constitutive manifold. To improve the convergence properties toward the global minima, Gebhardt et al.[9] proposed an *approximated nonlinear optimization problem* (aNLP) that accelerates the initialization by relying on local reconstructions of the constitutive manifold. Later on, the same principle has been extended toward the dynamic case in [10] and also to deal with contact problems that are formulated as a *mathematical program with complementarity constraints* (MPCC) in [8].

In addition to all these contributions dealing with the solution of the DDCM problem, there are also some efforts focusing on the existence of solutions. For instance, Conti et al.[3] prove, in the small deformation setting, the existence of solutions for the data-driven elasticity problem. Such an important result relies on a transversality argument that deals with both, the global material data set (i.e., the strain–stress fields that locally look like elements of the measured data sets), and the material-independent set (i.e., strain–stress fields that satisfy in a strong sense the equilibrium and compatibility conditions as well as the essential and natural boundary conditions). Later on, Conti et al.[4] prove, in the finite deformation setting, the existence of solutions, but this time relying only on some type of coercitivity of the material data set. To the best of our knowledge, that last result is the strongest result for DDCM up to date. However, the properties of data-driven elasticity problems that are discretized into finite elements are still not well understood, and this is exactly our entry point.

In this work, we systematically investigate the mathematical structure and numerical solution of data-driven elasticity problems defined on a closed interval of the real line, which are discretized in the sense of the finite element method. This one-dimensional setting, even when generalizable to higher dimension to some extent, allows us to gain a deeper understanding of the underlying DCQP in DDCM, either in its constrained form or its penalized variant. We therefore prove the global solvability for both of them. In addition, we also propose a new structure-specific initialization for a solution strategy relying on an ADM, and we prove that the initialization is already globally optimal under certain symmetries of the data set and the body forces. This is also accompanied with the corresponding error and convergence analysis for a case in which the analytic solution is well known.

The outline of this article is as follows. Section 2 provides the motivating problem, which relies on a one-dimensional continuous idealization that is afterward discretized by means of the finite element method. Then, we introduce the basic theory for solving finite-dimensional continuous optimization problems together with its specialization to two globally solvable optimization problems in DDCM. Furthermore, the structure of the solutions is mathematically analyzed in both cases. Then, we introduce a method to efficiently solve the problems formulated comprised by two main steps: *i*) a structure-specific initialization, followed by *ii*) an alternating direction method. Section 3 investigates the structure of uniform and nonuniform discretizations that are used to state the DDCM problems to be solved. By means of several examples of increasing difficulty, we then show the remarkable properties of the setting developed. Finally, in Sect. 4, we give a closure to the present work with a summary and concluding remarks.

## Problem formulations and analysis

### Motivating problem

Given a bounded domain $\Omega \subset \mathbb {R}$, consider a physical body that occupies the closure $\overline{\Omega }$ and is subjected to body forces $f \in L^2(\Omega )$. All the necessary conditions (in a weak sense) required to formulate the equilibrium state up to constitutive dependencies can be described with the help of: *three primal fields*, e.g., the position $u \in H^1_0(\Omega )$, the strain $e \in L^2(\Omega )$ and the stress $s \in L^2(\Omega )$; and *two dual fields* $\lambda \in H^1_0(\Omega )$ and $\mu \in L^2(\Omega )$, where the first one corresponds to the enforcement of the equilibrium in itself and the second one to the enforcement of the compatibility condition between the primal fields *u* and *e*. In this work, we consider only those conditions that can be weakly formulated as

1 $$\begin{aligned} \Phi ((\lambda , \mu ), (u, e, s); f)= 0 \quad \forall (\lambda , \mu ) \in H^1_0(\Omega ) \times L^2(\Omega ) \end{aligned}$$

with

2 $$\begin{aligned} \Phi ((\lambda , \mu ), (u, e, s); f):=\langle {\lambda '},{s}\rangle {_{L^2(\Omega )}} - \langle {\lambda },{f}\rangle {_{L^2(\Omega )}} + \langle {\mu },{e - u'}\rangle {_{L^2(\Omega )}}, \end{aligned}$$

where $\langle {}\cdot {},{}\cdot {}\rangle$ represents the $L^2$ scalar product and the operator $(\,\cdot \,)'$ is the weak differentiation. In the current particular context, we adopt for simplicity the setting of infinite-dimensional spaces equipped with inner products, i.e., Hilbert spaces. However, for more general formulations, we would need to adopt spaces with less structure, such as Sobolev spaces and thus, involving dual spaces and dual pairings. To include the constitutive dependencies, let us consider the finite index set $\mathcal {D}$ where $|{\mathcal {D}}|$ is the number of independent experimental measurements available and

3 $$\begin{aligned} \mathfrak {D}= \{(\mathfrak {e}^d, \mathfrak {s}^d)\}_{d \in \mathcal {D}} \end{aligned}$$

represents the set of all experimental pairs that lie, up to a certain tolerance, on the underlying constitutive manifold whose local or global representation is unknown. Here, we seek coupled *data fields* given by a discrete-valued function $z = (\tilde{e}, \tilde{s})$ in the set $Z = L^2(\Omega , \mathfrak {D})$. Finally, by choosing proximity measures induced by the $L^2$-norm with a positive scaling parameter *c* that provides unit consistency, a possible realization of the DDCM approach relies on solving an infinite-dimensional discrete–continuous quadratic optimization problem given in the weak form

4 $$\begin{aligned} \inf _{(u,e,s, \tilde{e},\tilde{s})} \ \frac{c}{2} \Vert {e - \tilde{e}}\Vert _{{L^2(\Omega )}}^2 + \frac{1}{2 c} \Vert {s - \tilde{s}}\Vert _{{L^2(\Omega )}}^2 \quad \text {s.t.}\quad \Phi ((\lambda , \mu ), (u, e, s); f)= 0 \quad \forall (\lambda , \mu ) . \end{aligned}$$

The corresponding saddle point formulation reads

5 $$\begin{aligned} \inf _{(x, z) \in X \times Z} \ \sup _{y \in Y} \ \frac{c}{2} \Vert {e - \tilde{e}}\Vert _{{L^2(\Omega )}}^2 + \frac{1}{2 c} \Vert {s - \tilde{s}}\Vert _{{L^2(\Omega )}}^2 + \Phi ((\lambda , \mu ), (u, e, s); f), \end{aligned}$$

where the primal field $x = (u, e, s)$ belongs to $X = H^1_0(\Omega ) \times L^2(\Omega ) \times L^2(\Omega )$ and the dual field $y = (\lambda , \mu )$ belongs to $Y = H^1_0(\Omega ) \times L^2(\Omega )$. The major difficulty in solving this problem is the discrete nature of the data field *z* which, because of the finiteness of $\mathfrak {D}$, necessarily has the form of a simple function

6 $$\begin{aligned} z(\omega ) = (\tilde{e},\tilde{s})(\omega ) = \sum _{n \in \mathcal {D}_a} \chi _{\Omega _n}(\omega ) (\mathfrak {e}^n, \mathfrak {s}^n) \quad \forall \omega \in \Omega . \end{aligned}$$

Herein, $\mathcal {D}_a \subseteq \mathcal {D}$ denotes the set of all *active* elements of $\mathfrak {D}$ (which are to be found subject to $\Phi$ vanishing identically), $\{\Omega _n\}_{n \in \mathcal {D}_a}$ is the associated partition of the domain $\Omega$ into measurable sets $\Omega _n$ (which is also to be sought simultaneously), and $\chi _{\Omega _n} :\Omega$ is the characteristic function of $\Omega _n$ defined as

7 $$\begin{aligned} \chi _{\Omega _n}(\omega ) :={\left\{ \begin{array}{ll} 1, &  \omega \in \Omega _n, \\ 0, &  \omega \notin \Omega _n. \end{array}\right. } \end{aligned}$$

To deal with (5), we are going to consider a spatial discretization by means of the finite element method. A finite element in $\mathbb {R}$ is a triplet $(K, P, \Sigma )$ where: *i*) *K* is a non-empty compact subset of $\mathbb {R}$; *ii*) *P* is a finite-dimensional vector space of functions on *K*; and *iii*) $\Sigma$ is a set of linear forms acting on the elements of *P* that are called degrees of freedom. Our spatial mesh $\mathcal {T}_h$ is then a set of $N_e$ compact elements $\{K_i\}_{i \in \mathcal {N}_e}$ with $\mathcal {N}_e =\{1, \dots , N_e\}$ such that

$$\begin{aligned} \overline{\Omega }= \bigcup _{i \in \mathcal {N}_e}K_i, \quad (K_i)^\circ \cap (K_j)^\circ = \emptyset \ \forall i \ne j, \quad \partial \Omega = \{a, b\} \text { with } b > a \text { and } {{\,\textrm{meas}\,}}(\Omega ) = b - a, \end{aligned}$$

where $(\,\cdot \,)^\circ$ represents the interior, $\partial (\,\cdot \,)$ the boundary, and ${{\,\textrm{meas}\,}}(\,\cdot \,)$ the measure of the set considered. Let *V*(*K*) be a Sobolev space associated with the element *K* and $\mathcal I_K :V(K) \rightarrow P$ be a local interpolation operator and $P = \mathbb {P}_k$ containing polynomials of degree up to *k*. Then, the following estimate holds [5]:

$$\begin{aligned} \Vert {v - \mathcal {I}_K(v)}\Vert _{{L^2(K)}} \le c_K h^{\ell +1}_K \Vert {v}\Vert _{{H^2(K)}}, \quad v \in V(K), \ \ell \le k, \end{aligned}$$

with $c_K$ being a constant, $h_K$ representing the diameter of the largest ball contained in *K*, and all partial derivatives assumed in the weak sense. The estimate can be extended to all of $\Omega$ as follows [5]:

$$\begin{aligned} \Vert {{v - \mathcal {I}_h(v)}}\Vert _{{{L^2(\Omega )}}} \le c_\Omega h^{\ell +1} \Vert {v}\Vert _{{H^2(\Omega )}}, \quad v \in V(\Omega ), \ \ell \le k, \end{aligned}$$

where $\mathcal {I}_h$ is the global interpolation operator, $c_\Omega$ is a constant, *h* is the largest $h_K$ in the mesh $\mathcal {T}_h$, and $V(\Omega )$ is the space in which the problem is formulated. For the current work, we introduce

8 $$\begin{aligned} V^0_h&:=\{v_h \in L^2(\Omega ):v_h|_{K_i} \in \mathbb {P}_0(K_i) \ \forall i \in \mathcal {N}_e\}, \end{aligned}$$

9 $$\begin{aligned} V^1_h&:=\{v_h \in H^1_0(\Omega ):v_h|_{K_i} \in \mathbb {P}_1(K_i) \ \forall i \in \mathcal {N}_e\}. \end{aligned}$$

The displacement field is going to be approximated within $V^1_h$ while the strain and the stress are going to be approximated within $V^0_h$, respectively. Analogously, the field $\lambda$ is going to be approximated within $V^1_h$ while the field $\mu$ is going to be approximated within $V^0_h$.

Now we consider $u \approx u_h = \sum _{i \in \mathcal {N}_n}u_i \phi _i$ and $\lambda \approx \lambda _h = \sum _{i \in \mathcal {N}_n}\lambda _i \phi _i$ with $u_i$ and $\lambda _i$ being nodal degrees of freedom, $\phi _i$ standing for the $i^\textrm{th}$ nodal shape function, and $\mathcal {N}_n = \{1, \dots , N_n\}$ where $N_n$ is the number of nodes. Similarly, $e \approx e_h = \sum _{i \in \mathcal {N}_e}e_i \chi _{K_i}$, $s \approx s_h = \sum _{i \in \mathcal {N}_e}s_i \chi _{K_i}$ and $\mu _h \approx \sum _{i \in \mathcal {N}_e}\mu _i \chi _{K_i}$ with $e_i$, $s_i$ and $\mu _i$ being elemental degrees of freedom and $\chi _{K_i}$ standing for the characteristic function corresponding to the $i^\textrm{th}$ element. We use $f \approx f_h = \sum _{i \in \mathcal {N}_e}f_i \chi _{K_i}$ with $f_i :={{\,\textrm{meas}\,}}(K_i)^{-1} \int _{K_i} f(x) \,dx$ for the body forces. Denote by $e, \tilde{e}, s, \tilde{s}, u, \lambda , \mu$ from now on the coefficient vectors. Then, we have

$$\begin{aligned} \frac{c}{2} \Vert {e_h - \tilde{e}_h}\Vert _{{L^2(\Omega )}}^2 + \frac{1}{2 c} \Vert {s_h - \tilde{s}_h}\Vert _{{L^2(\Omega )}}^2&= \frac{1}{2} \sum _{i \in \mathcal {N}_e}{{\,\textrm{meas}\,}}(K_i) \Bigl ( c (e_i - \tilde{e}_i)^2 + \frac{1}{c} (s_i - \tilde{s}_i)^2 \Bigr ) \\&= \frac{1}{2} (e - \tilde{e})^TW_e(e - \tilde{e}) + \frac{1}{2} (s - \tilde{s})^TW_s(s - \tilde{s}) \\&= \frac{1}{2} \Vert {e - \tilde{e}}\Vert _{{W_e}}^2 + \frac{1}{2} \Vert {s - \tilde{s}}\Vert _{{W_s}}^2 \end{aligned}$$

with positive definite diagonal weight matrices $W_e= c D$ and $W_s= \frac{1}{c} D$, where $D :={{\,\textrm{Diag}\,}}[{{\,\textrm{meas}\,}}(K_i)]$. Next, we obtain

$$\begin{aligned} \langle {\lambda _h'},{s_h}\rangle {_{L^2(\Omega )}} - \langle {\lambda _h},{f_h}\rangle {_{L^2(\Omega )}} + \langle {\mu _h},{e_h - u_h'}\rangle {_{L^2(\Omega )}}&= \\ \sum _{i \in \mathcal {N}_n}\lambda _i \biggl ( \sum _{j \in \mathcal {N}_e} \int _{K_j} \phi _i'(x) \,dx \, s_j - \sum _{j \in \mathcal {N}_e} \int _{K_j} \phi _i(x) \,dx \, f_j \biggr )&+ {} \\ \sum _{i \in \mathcal {N}_e} \mu _i \biggl ( \sum _{j \in \mathcal {N}_e} {{\,\textrm{meas}\,}}(K_i) \delta _{ij} e_j - \sum _{j \in \mathcal {N}_n} \int _{K_i} \phi _j'(x) \,dx \, u_j \biggr )&= \lambda ^T(B^Ts - C^Tf) + \mu ^T(D e - B u) \\&= \langle {\lambda },{B^Ts - C^Tf}\rangle {_{}} + \langle {\mu },{D e - B u}\rangle {_{}} \end{aligned}$$

with *B* and *C* injective and *D* as above. Moreover, the discretization of $\Omega$ by means of finite elements naturally introduces a domain decomposition that will be used to determine the sub-domain where the active experimental data are going to be associated with.

Finally, by introducing $q :=C^T f$ and scaling the strains *e* and the dual field $\mu$ to obtain $\varepsilon :=D e$, $W_\varepsilon :=D^{-1} W_eD^{-1} = W_s^{-1}$, and $\eta :=W_{\varepsilon }^{-1}\mu$, we arrive at the following spatial discretization of the saddle point problem (5):

10 $$\begin{aligned} \min _{(u,\varepsilon ,s, \tilde{\varepsilon },\tilde{s})} \ \max _{(\lambda , \eta )} \ \frac{1}{2} \Vert {\varepsilon - \tilde{\varepsilon }}\Vert _{{W_{\varepsilon }}}^2 + \frac{1}{2} \Vert {s - \tilde{s}}\Vert _{{W_s}}^2 +\, \langle {{\lambda }, B^Ts - q}\rangle \,+\, \langle {\eta },{\varepsilon - B u}\rangle {_{W_{\varepsilon }}}. \end{aligned}$$

Thus, in the remainder of this work, we are going to analyze the spatially discretized discrete–continuous quadratic optimization problem (DCQP) for DDCM in the compact form:

11 $$\begin{aligned} \begin{aligned} \smash [b]{\min _{(u,\varepsilon ,s, \tilde{\varepsilon },\tilde{s})} \ \frac{1}{2} \Vert {\varepsilon - \tilde{\varepsilon }}\Vert _{{W_\varepsilon }}^2 + \frac{1}{2} \Vert {s - \tilde{s}}\Vert _{{W_s}}^2} \quad \text {s.t.}\quad B^Ts - q&= 0, \\ \varepsilon - B u&= 0. \end{aligned} \end{aligned}$$

In the analysis, we will exploit the specific structure of this DCQP: The discrete variables $(\tilde{\varepsilon }, \tilde{s})$ appear only in the objective, which is a norm-square that measures the distance of $(\tilde{\varepsilon }, \tilde{s})$ to the continuous variables $(\varepsilon , s)$, and the constraints involve both *B* and $B^T$. We will also make use of the relation $W_\varepsilon = W_s^{-1}$. Further structural properties will be discussed below where we revert to the notation *e* for $\varepsilon$ and $\mu$ for $\eta$. Likewise, the notation $(\tilde{e}, \tilde{s})$ will stand for $(\tilde{\varepsilon }, \tilde{s})$ in the scaled product set $\mathfrak {D}_{\textrm{sc}}^m :=\{(D \tilde{e}, \tilde{s}):(\tilde{e}, \tilde{s}) \in \mathfrak {D}^m\}$.

#### Remark 1

Although the present formulation is originally intended for strain and stress measures that are induced by the one-dimensional idealization considered here, uni-axial experimental data obtained from three-dimensional test specimens can be used as well. For such an end, the argument of the integrals involved in the objective function and the balance equation have to be multiplied with the cross-sectional area (as a function of the length) as done, for instance, by Kirchdoerfer and Ortiz [13] and Kanno [11]. Upon re-scaling this kind of model, the form (11) is fully recovered, and thus, the remainder of this work does not require further adjustments. Note, however, that the mechanical model presented here needs still to be extended to two and three dimensions, which will of course be addressed in coming works.

### Basic QP theory

We begin with a summary of standard QP theory, which applies to the DCQP with *fixed* discrete variables. A finite-dimensional continuous optimization problem with quadratic objective and linear constraints is called a *quadratic optimization problem* (QP) and has the general form

$$\begin{aligned} \min _{x \in \mathbb {R}^n} \ \frac{1}{2} x^TH x + c^Tx \quad \text {s.t.}\quad A x = b, \quad C x \ge d, \end{aligned}$$

where $H \in \mathbb {R}^{n \times n}$ is symmetric and *A*, *b*, respectively, *C*, *d* are matrices and vectors with compatible dimensions. In this work we are only concerned with purely equality-constrained QPs (EQPs in brief) that are *convex* in addition, that is, *H* is positive semidefinite ($H \ge 0$) or even positive definite ($H > 0$). We define the *Lagrangian* of the general EQP with a positive sign for the multiplier vector *y*,

$$\begin{aligned} L(x, y) = \frac{1}{2} x^TH x + c^Tx + y^T(Ax - b) = \frac{1}{2} \Vert {x}\Vert _{{H}}^2 + \langle {c},{x}\rangle {_{}} + \langle {y},{Ax - b}\rangle {_{}}. \end{aligned}$$

Standard optimization theory then gives the following results, see, e.g., [16].

#### Proposition 1

Consider a convex EQP with $A \in \mathbb {R}^{m \times n}$, $b \in \mathbb {R}^m$. Then:

1. Every minimizer is a global minimizer.
2. The set of all minimizers is an affine subspace of $\mathbb {R}^n$.
3. Given a minimizer $x^*$, there exists a multiplier $y^* \in \mathbb {R}^m$ such that the first-order necessary optimality conditions *(KKT conditions)* hold,
  $$\begin{aligned} 0 = \partial _x L(x^*, y^*)&= H x^* + c + A^Ty^*, \\ 0 = \partial _y L(x^*, y^*)&= A x^* - b, \end{aligned}$$
  equivalently the (linear-indefinite) *KKT system*
  $$\begin{aligned} \left[ \begin{array}{cc} H & \quad A^T\\ A \end{array}\right] \left( \begin{array}{cc} x^* \\ y^* \end{array}\right) = \left( \begin{array}{cc} -c \\ b. \end{array}\right) \end{aligned}$$
  In particular, $x^*$ and $y^*$ depend linearly on *b* and *c*.
4. The necessary conditions are also sufficient in the (given) convex case.
5. If $H > 0$ on the kernel of *A*, then the primal solution $x^*$ is unique and hence a strict minimizer.
6. If *A* has full row rank, ${{\,\textrm{rank}\,}}A = m \le n$, then the dual solution $y^*$ is unique.
7. If both (5) and (6) hold, then the *KKT matrix* in (3) is nonsingular. and the unique *KKT point* $(x^*, y^*)$ is determined as follows. Given any null space basis matrix $Z \in \mathbb {R}^{(n - m) \times n}$ of *A* and any matrix $Y \in \mathbb {R}^{m \times n}$ with linearly independent columns such that $$\left[ \begin{array}{cc} Y&Z\end{array}\right] \in \mathbb {R}^{n \times n}$$ is nonsingular, then:
  - the *projected Hessian* $Z^TH Z$ is positive definite;
  - the matrix $A Y \in \mathbb {R}^{m \times m}$ is nonsingular;
  - $x^* = Y x_1^* + Z x_2^*$ where $x_1^*$ represents a particular solution of $A x = b$ and $x_2^*$ optimizes over the remaining degrees of freedom,
    $$\begin{aligned} x_1^* = (A Y)^{-1} b, \quad x_2^* = -(Z^TH Z)^{-1} Z^T(H Y x_1^* + c); \end{aligned}$$
  - $$y^* = -(A Y)^{-T} Y^T(H x^* + c)$$.

The structural relation between our DCQP and the associated QP is provided by the following result.

#### Proposition 2

Given the DCQP

$$\begin{aligned} \min _{(x,z) \in \mathbb {R}^n \times \bar{\mathfrak {D}}} \ \frac{1}{2} \Vert {W}\Vert _{{}}{P x - z}^2 \quad \text {s.t.}\quad A x = b, \end{aligned}$$

with a finite set $\bar{\mathfrak {D}}$, let

$$\begin{aligned} H = P^TW P, \quad K = \left[ \begin{array}{cc} H & \quad A^T\\ A \end{array}\right] , \quad R = \left[ \begin{array}{cc} P^TW \\ &  I \end{array}\right] . \end{aligned}$$

If *K* is nonsingular, then every fixed $z \in \bar{\mathfrak {D}}$ admits the unique EQP minimizer and unique multiplier

$$\begin{aligned} x^*(z) = \left[ \begin{array}{cc} I&\quad 0 \end{array}\right] K^{-1} R \left( \begin{array}{cc} z \\ b \end{array}\right) \quad \text {and}\quad y^*(z) = \left[ \begin{array}{cc} 0&\quad I\end{array}\right] K^{-1} R \left( \begin{array}{c} z \\ b \end{array}\right) , \end{aligned}$$

and all global solutions of the discrete–continuous optimization problem satisfy

12 $$\begin{aligned} z^* \in \textrm{argmin}_{z \in \bar{\mathfrak {D}}} \Vert P x^*(z) - z\Vert _2^2. \end{aligned}$$

In theory, we thus find all global solutions by computing $K^{-1}$ and comparing finitely many norm-squares of linear functions of the discrete variables *z* (and of the input *b*).

#### Proof

This follows from items (1)–(6) of the preceding proposition with the finiteness of $\bar{\mathfrak {D}}$.

#### Remark 2

The seemingly strong assumption of a nonsingular KKT matrix *K* is always satisfied in the problems studied below. For this reason, we refrain from presenting a weaker analogon of Proposition 2, even though the uniqueness of multipliers is not exploited in our study.

#### Remark 3

For the global solvability, it suffices that

- the set $\bar{\mathfrak {D}}$ is finite and
- every fixed discrete variable $z \in \bar{\mathfrak {D}}$ admits a unique global minimizer $x^*(z)$ of the reduced problem.

For algorithmic tractability, we need that

- evaluating $x^*(z)$ is tractable and
- minimizing the resulting objective over $\bar{\mathfrak {D}}$ is tractable.

Unfortunately, $\bar{\mathfrak {D}}$ is actually the astronomically large product set $\mathfrak {D}^m$ in our setting, and solving the DCQP by *enumeration* (i.e., comparing all possible values) is not feasible even though $x^*(z)$ is easily computed. The sketched solution approach works only if further structural properties yield a sufficiently small subset of $\mathfrak {D}^m$ that is guaranteed to contain a global solution and that can be computed efficiently.

### Optimization problems in DDCM

In this section, we provide a complete structural analysis of the DCQP (11) and of an associated penalty formulation.

#### Definition

For a proper geometric interpretation of the following results, we need the pair of dual spaces

$$\begin{aligned} \mathbb {R}^m_e:=(\mathbb {R}^m, \langle {}\cdot {},{}\cdot {}\rangle {_{W_e}}) \quad {and} \quad \mathbb {R}^m_s:=(\mathbb {R}^m,\langle {}\cdot {},{}\cdot {}\rangle {_{W_s}}) \end{aligned}$$

i.e., $\mathbb {R}^m$ with the appropriate scalar products for (scaled) strains *e* and stresses *s*, where $W_s^{-1}= W_e$.

We also need the *Moore–Penrose inverse* (or simply *pseudoinverse*) in a slightly generalized setting; see [1, 17]. Given Euclidean spaces *X*, *Y* with scalar products $\langle {}\cdot {},{}\cdot {}\rangle _X$, respectively, $\langle {}\cdot {},{}\cdot {}\rangle _Y$ and a linear map $A:X \rightarrow Y$, the adjoint of *A* is the unique linear map $A^*:Y \rightarrow X$ that satisfies

$$\begin{aligned} \langle {x},{A^* y}\rangle {_{X}} = \langle {A x},{y}\rangle {_{Y}} \quad \forall (x, y) \in X \times Y, \end{aligned}$$

and the pseudoinverse of *A* is the unique linear map $A^+:Y \rightarrow X$ that satisfies the four Penrose axioms,

$$\begin{aligned} A A^+ A&= A,&A^+ A A^+&= A^+,&(A A^+)^*&= A A^+,&(A^+ A)^*&= A^+ A. \end{aligned}$$

From the self-adjoint maps in the two last axioms, one immediately obtains the orthogonal projections onto the null space of *A* in *X*, the image space of *A* in *Y*, and onto their orthogonal complements,

$$\begin{aligned} I - A^+ A&= P_{\ker A}: X \rightarrow (\ker A),&A^+ A&= P_{\ker A}^\perp , \\ A A^+&= P_{\text {im}A}: Y \rightarrow \text {im}A,&I - A A^+&= P_{\text {im}A}^\perp . \end{aligned}$$

If *A* is injective (or surjective), then $A^+$ is a left inverse (or right inverse) with explicit representation

$$\begin{aligned} A^+&= (A^* A)^{-1} A^* \quad \text{(A } \text{ injective) }, \\ A^+&= A^* (A A^*)^{-1} \quad \text{(A } \text{ surjective) }. \end{aligned}$$

In matrix representation with respect to the canonical bases of $\mathbb {R}^m$ and $\mathbb {R}^n$, our specific linear maps $B:\mathbb {R}^n \rightarrow \mathbb {R}^m_e$ (injective) and $B^T:\mathbb {R}^m_s\rightarrow \mathbb {R}^n$ (surjective) have respective adjoints and pseudoinverses

$$\begin{aligned} B^*&= B^TW_e,&B^+&= (B^TW_eB)^{-1} B^TW_e, \\ (B^T)^*&= W_s^{-1}B,&(B^T)^+&= W_s^{-1}B (B^TW_s^{-1}B)^{-1}. \end{aligned}$$

With these preparations, we now analyze the general DCQP and its associated penalty formulation. Recall that the discrete variables live in a scaled product set, $(\tilde{e}, \tilde{s}) \in \mathfrak {D}_{\textrm{sc}}^m = \{(D \tilde{e}, \tilde{s}):(\tilde{e}, \tilde{s}) \in \mathfrak {D}^m\}$.

#### Problem

(**P1**) Given a matrix $B \in \mathbb {R}^{m \times n}$ (injective, i.e., ${{\,\textrm{rank}\,}}B = n \le m$), a vector $q \in \mathbb {R}^n$, diagonal weight matrices $W_e, W_s> 0$ in $\mathbb {R}^{m \times m}$, and the data set $\mathfrak {D}= \{(\mathfrak {e}^d, \mathfrak {s}^d)\}_{d \in \mathcal {D}}$, a discrete–continuous optimization problem for finding $(u, e, s) \in \mathbb {R}^n \times \mathbb {R}^m_e\times \mathbb {R}^m_s$ and $(\tilde{e}, \tilde{s}) \in \mathfrak {D}_{\textrm{sc}}^m$ can be stated as

13 $$\begin{aligned} \begin{aligned} \smash [b]{\min _{(u,e,s,\tilde{e},\tilde{s})} \ \frac{1}{2} \Vert {e - \tilde{e}}\Vert _{{W_e}}^2 + \frac{1}{2} \Vert {s - \tilde{s}}\Vert _{{W_s}}^2} \quad \text {s.t.}\quad B^Ts - q&= 0, \\ e - B u&= 0. \end{aligned} \end{aligned}$$

#### Proposition 3

P1 is globally solvable. Every optimal pair of discrete variables $(\tilde{e}^*, \tilde{s}^*)$ minimizes the norm-square $\Vert {P_{\text {im}B}^\perp \tilde{e}}\Vert _{{W_e}}^2 + \Vert {P_{\ker B^T}^\perp \tilde{s}- (B^T)^+ q}\Vert _{{W_s}}^2$ over $(\tilde{e}, \tilde{s}) \in \mathfrak {D}_{\textrm{sc}}^m$.

#### Proof

Fixing the discrete variables $(\tilde{e}, \tilde{s}) \in \mathfrak {D}_{\textrm{sc}}^m$, we obtain the EQP

14 $$\begin{aligned} \begin{aligned} \smash [b]{\min _{(u,e,s)} \ \frac{1}{2} \Vert {e - \tilde{e}}\Vert _{{W_e}}^2 + \frac{1}{2} \Vert {s - \tilde{s}}\Vert _{{W_s}}^2} \quad \text {s.t.}\quad B^Ts - q&= 0, \\ e - B u&= 0 \end{aligned} \end{aligned}$$

with dual variables $\lambda \in \mathbb {R}^n$, $\mu \in \mathbb {R}^m_e$ and Lagrangian

$$\begin{aligned} L(u, e, s, \lambda , \mu ) = \frac{1}{2} \Vert {e - \tilde{e}}\Vert _{{W_e}}^2 + \frac{1}{2} \Vert {s - \tilde{s}}\Vert _{{W_s}}^2 + \langle {B^Ts - q},{+}\rangle {_{\lambda }} \langle {\mu },{e - B u}\rangle {_{W_e}}. \end{aligned}$$

We observe that, by fixing $(\tilde{e}, \tilde{s})$, problem P1 separates into two independent convex EQPs,

$$\begin{aligned} \min _{(u,e)} \ \frac{1}{2} \Vert {e - \tilde{e}}\Vert _{{W_e}}^2 \quad \text {s.t.}\quad e - B u = 0 \end{aligned}$$

and

$$\begin{aligned} \min _s \ \frac{1}{2} \Vert {s - \tilde{s}}\Vert _{{W_s}}^2 \quad \text {s.t.}\quad B^Ts - q = 0. \end{aligned}$$

The associated KKT systems are

$$\begin{aligned} \left[ \begin{array}{ccc} 0 & \quad & \quad -B^T\\ & \quad W_e& \quad I \\ -B & \quad I & \quad 0 \end{array}\right] \left( \begin{array}{c} u \\ e \\ W_e\mu \end{array}\right) = \left( \begin{array}{c} 0 \\ W_e\tilde{e}\\ 0 \end{array}\right) \quad \text {and}\quad \left[ \begin{array}{cc} W_s& \quad B \\ B^T\end{array}\right] \left( \begin{array}{c} s \\ \lambda \end{array}\right) = \left( \begin{array}{c} W_s\tilde{s}\\ q \end{array}\right) . \end{aligned}$$

The first KKT matrix is nonsingular with $$A = \left[ \begin{array}{cc}-B&I \end{array}\right] $$ of full row rank *m* and $Z^TH Z = B^TW_eB > 0$ in $\mathbb {R}^{n \times n}$ where $$Z^T= \left[ \begin{array}{cc}I&B^T\end{array}\right] $$. The unique global minimizer and the unique multiplier of the first EQP are then easily obtained in terms of the pseudoinverse $B^+$, the $W_e$-orthogonal projection $P_{\text {im}B}= B B^+$, and the complementary projection $P_{\text {im}B}^\perp = I - B B^+$,

$$\begin{aligned} (u^*, e^*, \mu ^*) = (B^+ \tilde{e}, BB^+ \tilde{e}, \tilde{e}- e^*) = (B^+ \tilde{e}, P_{\text {im}B}\tilde{e}, P_{\text {im}B}^\perp \tilde{e}) . \end{aligned}$$

We thus have the $W_e$-orthogonal decomposition $\tilde{e}= e^* + \mu ^* \in \text {im}B\oplus (\text {im}B)^\perp$, and the optimal value is

$$\begin{aligned} \frac{1}{2} \Vert {e^* - \tilde{e}}\Vert _{{W_e}}^2 = \frac{1}{2} \Vert {\mu ^*}\Vert _{{W_e}}^2 = \frac{1}{2} \Vert {P_{\text {im}B}^\perp \tilde{e}}\Vert _{{W_e}}^2 . \end{aligned}$$

The second KKT matrix is also nonsingular with $H = W_s> 0$ and $A = B^T$ of full row rank *n*. Here, the unique global minimizer and the unique multiplier are obtained in terms of the pseudoinverse $(B^T)^+$ and the $W_s$-orthogonal projection $P_{\ker B^T}= I - (B^T)^+ B^T$,

$$\begin{aligned} s^*&= \tilde{s}- (B^T)^+ (B^T\tilde{s}- q) = P_{\ker B^T}\tilde{s}+ (B^T)^+ q, \\ \lambda ^*&= (B^TW_s^{-1}B)^{-1} (B^T\tilde{s}- q). \end{aligned}$$

The optimal value becomes

$$\begin{aligned} \frac{1}{2} \Vert {s^* - \tilde{s}}\Vert _{{W_s}}^2 = \frac{1}{2} \Vert {(B^T)^+ (B^T\tilde{s}- q)}\Vert _{{W_s}}^2 = \frac{1}{2} \Vert {P_{\ker B^T}^\perp \tilde{s}- (B^T)^+ q}\Vert _{{W_s}}^2 . \end{aligned}$$

In summary, the EQP (14) admits the unique global minimizer $(u^*, e^*, s^*)$ with unique multipliers $(\lambda ^*, \mu ^*)$ where

$$\begin{aligned} (u^*, e^*, s^*, \lambda ^*, \mu ^*) = (B^+ \tilde{e}, P_{\text {im}B}\tilde{e}, P_{\ker B^T}\tilde{s}+ (B^T)^+ q, (B^TW_s^{-1}B)^{-1} (B^T\tilde{s}- q), P_{\text {im}B}^\perp \tilde{e}). \end{aligned}$$

By (12), there exists a subset $\mathfrak {D}_{\textrm{sc}}^* \subseteq \mathfrak {D}_{\textrm{sc}}^m$ (in general not a product set) such that every data pair $(\tilde{e}^*, \tilde{s}^*) \in \mathfrak {D}_{\textrm{sc}}^*$ together with the associated EQP solution $(u^*, e^*, s^*)$ yields the smallest value of (14) over all $(\tilde{e}, \tilde{s}) \in \mathfrak {D}_{\textrm{sc}}^m$:

$$\begin{aligned} \frac{1}{2} \Vert {e^* - \tilde{e}^*}\Vert _{{W_e}}^2 + \frac{1}{2} \Vert {s^* - \tilde{s}^*}\Vert _{{W_s}}^2 = \min _{(\tilde{e}, \tilde{s}) \in \mathfrak {D}_{\textrm{sc}}^m} \frac{1}{2} \Vert {P_{\text {im}B}^\perp \tilde{e}}\Vert _{{W_e}}^2 + \frac{1}{2} \Vert {P_{\ker B^T}^\perp \tilde{s}- (B^T)^+ q}\Vert _{{W_s}}^2 . \end{aligned}$$

The global solution set of P1 is thus

$$\begin{aligned} \{(u^*, e^*, s^*, \tilde{e}^*, \tilde{s}^*):(\tilde{e}^*, \tilde{s}^*) \in \mathfrak {D}_{\textrm{sc}}^*\}. \end{aligned}$$

This completes the proof.

#### Problem

(**P2**) Given the injective matrix *B*, vector *q*, weight matrices $W_e, W_s$, and the data set $\mathfrak {D}$ of P1 and parameters $\alpha , \beta \in \mathbb {R}_{>0}$, a discrete–continuous quadratic penalty formulation of P1 for finding $(u, e, s) \in \mathbb {R}^n \times \mathbb {R}^m_e\times \mathbb {R}^m_s$ and $(\tilde{e}, \tilde{s}) \in \mathfrak {D}_{\textrm{sc}}^m$ can be stated as

15 $$\begin{aligned} \min _{(u,e,s,\tilde{e},\tilde{s})} \ \frac{1}{2} \Vert {e - \tilde{e}}\Vert _{{W_e}}^2 + \frac{1}{2} \Vert {s - \tilde{s}}\Vert _{{W_s}}^2 + \frac{\alpha }{2} \Vert B^Ts - q\Vert _2^2 + \frac{\beta }{2} \Vert {e - B u}\Vert _{{W_e}}^2. \end{aligned}$$

#### Proposition 4

P2 is globally solvable with continuous variables $(u^*, e_\beta ^*, s_\alpha ^*)$. Every optimal pair of discrete variables $(\tilde{e}^*, \tilde{s}^*)$ minimizes the norm-square $\frac{\beta }{1 + \beta } \Vert {P_{\text {im}B}^\perp \tilde{e}}\Vert _{{W_e}}^2 + \Vert {B^T\tilde{s}- q}\Vert _{{(\alpha ^{-1}I + B^TW_s^{-1}B)^{-1}}}^2$.

#### Proof

Fixing the discrete variables $(\tilde{e}, \tilde{s}) \in \mathfrak {D}_{\textrm{sc}}^m$ and minimizing (15) over (*u*, *e*, *s*) yields the optimality conditions

$$\begin{aligned} -\beta B^TW_e(e - Bu)&= 0, \\ W_e(e - \tilde{e}) + \beta W_e(e - B u)&= 0, \\ W_s(s - \tilde{s}) + \alpha B (B^Ts - q)&= 0, \end{aligned}$$

with equivalent matrix form

$$\begin{aligned} \left[ \begin{array}{ccc} \beta B^TW_eB & \quad -\beta B^TW_e\\ -\beta W_eB & \quad (1 + \beta ) W_e\\ & \quad & \quad W_s+ \alpha B B^T\end{array}\right] \left( \begin{array}{c} u \\ e \\ s \end{array}\right) = \left( \begin{array}{c}0 \\ W_e\tilde{e}\\ W_s\tilde{s}+ \alpha B q \end{array}\right) . \end{aligned}$$

Block elimination with pivot $(1 + \beta ) W_e$ shows that the upper left two by two submatrix is positive definite. Using again the Moore–Penrose pseudoinverse $B^+ = (B^TW_eB)^{-1} B^TW_e$, the projection $P_{\text {im}B}= B B^+$, and matrices $T_\beta = P_{\text {im}B}+ (1 + \beta ) P_{\text {im}B}^\perp$ (with $T_\beta ^{-1}= P_{\text {im}B}+ (1 + \beta )^{-1} P_{\text {im}B}^\perp$) and $S_\alpha = W_s+ \alpha B B^T$ (which is positive definite), the unique solution evaluates to

$$\begin{aligned} (u^*, e_\beta ^*, s_\alpha ^*) = (B^+ \tilde{e}, T_\beta ^{-1}\tilde{e}, S_\alpha ^{-1}(W_s\tilde{s}+ \alpha B q)) . \end{aligned}$$

Substituting this solution into (15) and minimizing over $(\tilde{e}, \tilde{s})$, in the finite set $\mathfrak {D}_{\textrm{sc}}^m$ gives again a subset $\mathfrak {D}_{\textrm{sc}}^* \subseteq \mathfrak {D}_{\textrm{sc}}^m$ such that every pair of discrete variables $(\tilde{e}^*, \tilde{s}^*) \in \mathfrak {D}_{\textrm{sc}}^*$ gives a global solution of P2. It remains to compute the objective value for $(u^*, e_\beta ^*, s_\alpha ^*)$. We start with

$$\begin{aligned} e_\beta ^*- \tilde{e}&= (T_\beta ^{-1}- I) \tilde{e}= ((1 + \beta )^{-1} P_{\text {im}B}^\perp - P_{\text {im}B}^\perp ) \tilde{e}= -\frac{\beta }{1 + \beta } P_{\text {im}B}^\perp \tilde{e}, \\ e_\beta ^*- B u^*&= (T_\beta ^{-1}- B B^+) \tilde{e}= (T_\beta ^{-1}- P_{\text {im}B}) \tilde{e}= \frac{1}{1 + \beta } P_{\text {im}B}^\perp \tilde{e}, \end{aligned}$$

which gives the $\beta$-dependent objective terms

$$\begin{aligned} \frac{1}{2} \Vert {e_\beta ^*- \tilde{e}}\Vert _{{W_e}}^2 + \frac{\beta }{2} \Vert {e_\beta ^*- B u^*}\Vert _{{W_e}}^2 = \frac{\beta }{2 (1 + \beta )} \Vert {P_{\text {im}B}^\perp \tilde{e}}\Vert _{{W_e}}^2. \end{aligned}$$

Using $S_\alpha ^{-1}= (I + \alpha W_s^{-1}B B^T)^{-1} W_s^{-1}$ and the matrix inversion lemma (also known as the Sherman–Morrison–Woodbury formula), we obtain the intermediate results

$$\begin{aligned} S_\alpha ^{-1}W_s\tilde{s}&= (I + \alpha W_s^{-1}B B^T)^{-1} \tilde{s}\\&= [I - W_s^{-1}B (\alpha ^{-1}I + B^TW_s^{-1}B)^{-1} B^T] \tilde{s}, \\ S_\alpha ^{-1}B q&= [I - W_s^{-1}B (\alpha ^{-1}I + B^TW_s^{-1}B)^{-1} B^T] W_s^{-1}B q \\&= W_s^{-1}B (\alpha ^{-1}I + B^TW_s^{-1}B)^{-1} [(\alpha ^{-1}I + B^TW_s^{-1}B) - B^TW_s^{-1}B] q \\&= \alpha ^{-1}W_s^{-1}B (\alpha ^{-1}I + B^TW_s^{-1}B)^{-1} q, \\ s_\alpha ^*&= \tilde{s}- W_s^{-1}B (\alpha ^{-1}I + B^TW_s^{-1}B)^{-1} (B^T\tilde{s}- q), \\ B^Ts_\alpha ^*- q&= B^T\tilde{s}- q - B^TW_s^{-1}B (\alpha ^{-1}I + B^TW_s^{-1}B)^{-1} (B^T\tilde{s}- q) \\&= [(\alpha ^{-1}I + B^TW_s^{-1}B) - B^TW_s^{-1}B] (\alpha ^{-1}I + B^TW_s^{-1}B)^{-1} (B^T\tilde{s}- q) \\&= \alpha ^{-1}(\alpha ^{-1}I + B^TW_s^{-1}B)^{-1} (B^T\tilde{s}- q). \end{aligned}$$

Setting $V_\alpha :=(\alpha ^{-1}I + B^TW_s^{-1}B)^{-1}$, this gives

$$\begin{aligned} s_\alpha ^*- \tilde{s}&= -W_s^{-1}B V_\alpha (B^T\tilde{s}- q), \\ B^Ts_\alpha ^*- q&= \alpha ^{-1}V_\alpha (B^T\tilde{s}- q). \end{aligned}$$

For the $\alpha$-dependent terms, we thus obtain

$$\begin{aligned} \frac{1}{2} \Vert {s_\alpha ^*- \tilde{s}}\Vert _{{W_s}}^2 + \frac{\alpha }{2} \Vert {B^Ts_\alpha ^*- q}\Vert _2^2&= \frac{1}{2} (B^T\tilde{s}- q)^TV_\alpha [B^TW_s^{-1}B + \alpha ^{-1}I] V_\alpha (B^T\tilde{s}- q) \\&= \frac{1}{2} \Vert {B^T\tilde{s}- q}\Vert _{{V_\alpha }}^2. \end{aligned}$$

The optimal value of (15) for fixed $(\tilde{e}, \tilde{s})$ finally reads

$$\begin{aligned} \frac{\beta }{2 (1 + \beta )} \Vert {P_{\text {im}B}^\perp \tilde{e}}\Vert _{{W_e}}^2 + \frac{1}{2} \Vert {B^T\tilde{s}- q}\Vert _{{V_\alpha }}^2. \end{aligned}$$

This completes the proof.

#### Remark 4

For $\alpha , \beta \rightarrow \infty$, the optimal value of P2 converges to the optimal value of P1 since $\beta / (1 + \beta )$ converges to one and $V_\alpha$ converges to $(B^TW_s^{-1}B)^{-1} = ((B^T)^+)^TW_s(B^T)^+$. As one should expect, the solutions of P2 converge to the solutions of P1 as well. For $e_\beta ^*$, this follows from $\lim _{\beta \rightarrow \infty } T_\beta ^{-1}= P_{\text {im}B}$ and for $s_\alpha ^*$ it follows from the intermediate result above:

$$\begin{aligned} \lim _{\alpha \rightarrow \infty } s_\alpha ^*&= \tilde{s}- W_s^{-1}B (B^TW_s^{-1}B)^{-1} (B^T\tilde{s}- q) \\&= \tilde{s}- (B^T)^+ (B^T\tilde{s}- q) = P_{\ker B^T}\tilde{s}+ (B^T)^+ q. \end{aligned}$$

Finally, $u^* = B^+ \tilde{e}$ has the same value as in P1.

### Alternating direction method

We have seen that fixing the discrete variables $(\tilde{e}, \tilde{s})$ reduces the DCQP to a standard convex QP whose unique solution yields optimal continuous variables $(u^*, e^*, s^*)$. Conversely, fixing (*e*, *s*) and possibly *u* leads to optimal discrete variables $(\tilde{e}^*, \tilde{s}^*)$ simply by minimizing the objective

16 $$\begin{aligned} \frac{1}{2} \Vert {e - \tilde{e}}\Vert _{{We}}^2 + \frac{1}{2} \Vert {s - \tilde{s}}\Vert _{{Ws}}^2 = \frac{1}{2} \sum _{i = 1}^m [ c D^{-1}_i (e_i - \tilde{e}_i)^2 + c^{-1} D_i (s_i - \tilde{s}_i)^2 ]. \end{aligned}$$

This is trivial and highly efficient even for large data sets and fine discretizations as it can be done component-wise, with complexity at most $O(m |{\mathfrak {D}}|)$ instead of $O(|{\mathfrak {D}}|^m)$.

Thus, we are in the setting for an ADM: We face a hard optimization problem whose complete set of variables, in our case (*x*, *z*), splits into two subsets, $x \in X$ and $z \in \mathfrak {D}_{\textrm{sc}}^m$, such that fixing one set yields an easy problem for the other set, which can even be solved to global optimality in our case. The ADM approach then fixes each variable set in turn to compute associated optimal values of the other set, so that one iteration on the complete variable vector consists in solving each of the two subproblems.

The ADM has originally been developed in connection with splitting methods and numerical solution techniques for partial differential equations and variational problems [7]. It works particularly well for convex problems since convergence to global minimizers can be enforced. In this context, a standard enhancement of the ADM is the *alternating direction method of multipliers* (ADMM) which employs an augmented Lagrangian approach and hence dual variables to speed up convergence. These and other splitting techniques for convex optimization problems are discussed in various sources; see, e.g., [2] or [6]. For discrete–continuous problems, though non-convex, the ADM is also the method of choice because typically it works well in practice. However, it will generally not converge to a local or global minimizer even if each subproblem is solved to global optimality (as in our case).

A crucial observation is that our structural analysis suggests a suitable initialization: By Proposition 3, we know that globally optimal discrete variables $z^* = (\tilde{e}^*, \tilde{s}^*)$ minimize the norm-square

17 $$\begin{aligned} \Vert {P_{\text {im}B}^\perp \tilde{e}}\Vert _{{W_e}}^2 + \Vert {P_{\ker B^T}^\perp \tilde{s}- (B^T)^+ q}\Vert _{{W_s}}^2 \end{aligned}$$

over $(\tilde{e}, \tilde{s}) \in \mathfrak {D}_{\textrm{sc}}^m$. This problem can be solved approximately by observing that $P_{\text {im}B}^\perp$ projects onto a subspace of small dimension in $\mathbb {R}^m_e$ while $P_{\ker B^T}^\perp$ projects onto a subspace of large dimension in $\mathbb {R}^m_s$. Indeed, we have $m > n$ and in case of the one-dimensional finite element discretization actually $m = n + 1$, so that $\dim (\text {im}B)^\perp = 1 \ll n = \dim (\ker B^T)^\perp$. This suggests to approximate (17) with the following objective for which a global minimizer can be computed efficiently:

$$\begin{aligned} \Vert {\tilde{s}- (B^T)^+ q}\Vert _{{W_s}}. \end{aligned}$$

Consequently, we initialize the ADM for solving P1 as follows.

1. Compute the unique minimum-norm solution $s_q$ of the underdetermined linear system $B^Ts = q$: $s_q = (B^T)^+ q \in (\ker B^T)^\perp$;
2. compute $(\tilde{e}_0, \tilde{s}_0) \in \text {argmin}_{(\tilde{e},\tilde{s}) \in \mathfrak {D}_{\textrm{sc}}^m} \Vert {\tilde{s}- s_q}\Vert _{{W_s}}$ (not unique in general) by *component-wise* minimization, $(\tilde{e}_{0i}, \tilde{s}_{0i}) \in \text {argmin}_{(\mathfrak {e},\mathfrak {s}) \in \mathfrak {D}} |{\mathfrak {s}- s_{qi}}|$ for $i = 1, \dots , m$.

At every iteration for $k = 0, 1, \dots$, we then first compute $(u_k, e_k, s_k)$ by solving the EQP with fixed $(\tilde{e}_k, \tilde{s}_k)$, and second we compute $(\tilde{e}_{k+1}, \tilde{s}_{k+1})$ by minimizing the objective with fixed $(u_k, e_k, s_k)$. The iteration terminates with $(u_k, e_k, s_k, \tilde{e}_k, \tilde{s}_k)$ when $(\tilde{e}_{k+1}, \tilde{s}_{k+1}) = (\tilde{e}_k, \tilde{s}_k)$. In this situation, the ADM has converged in the strict mathematical sense that all further iterates would coincide with $(u_k, e_k, s_k, \tilde{e}_k, \tilde{s}_k)$. The ADM proposed originally by Kirchdoerfer and Ortiz [13, Algorithm 1] initializes $(\tilde{e}_0, \tilde{s}_0)$ randomly from $\mathfrak {D}_{\textrm{sc}}^m$.

We conclude this section with a condition that implies global optimality of our initialization. Below we will see that an important class of practical problems actually satisfies that condition.

#### Proposition 5

Suppose that

1. $$(\tilde{e}^*, \tilde{s}^*) \in \text {im}B \times (\ker B^T)^\perp ;$$
2. $$\Vert {\tilde{s}^* - s_q}\Vert _{{W_s}} = \min _{(\tilde{e}, \tilde{s}) \in \mathfrak {D}_{\textrm{sc}}^m} \Vert {\tilde{s}- s_q}\Vert _{{W_s}}$$.

Then, $(\tilde{e}^*, \tilde{s}^*)$ is globally optimal for problem P1.

#### Proof

Assumption (1) implies $\Vert {P_{\text {im}B}^\perp \tilde{e}^*}\Vert _{{W_e}}^2 + \Vert {P_{\ker B^T}^\perp \tilde{s}^* - s_q}\Vert _{{W_s}}^2 = 0 + \Vert {\tilde{s}^* - s_q}\Vert _{{W_s}}^2$ with the second term being globally optimal by assumption (2). The claim now follows from Proposition 3.

## Computational results

To substantiate the structural analysis developed in Section 2, we will perform three types of numerical experiments, two of them with a finite element discretization of a one-dimensional elastic body and the third one with a truss structure that has been studied in [11]. In preparation for these experiments, we first present details of the discretized problem from Section 2.1 and then give an overview of the strain–stress data sets that we use. The numerical computations are performed on an AMD Ryzen 7 7700 8-Core CPU with 64GiB RAM running under the GNU/Linux operating system.

### Discretized problem

In the simplest case, we consider an elastic body that is uniformly discretized into *m* finite elements $K_i = [x_{i-1}, x_i]$ with $m + 1 = n + 2$ nodes $0 = x_0< \dots < x_{n+1} = L$, where the equation $\varepsilon = \bar{B} \bar{u}$ approximates the differential equation $e = u'$ by central differences with the surjective matrix

$$\begin{aligned} \bar{B} = \left[ \begin{array}{cccc} -1 &  1 \\   &  \ddots &  \ddots \\   &  &  -1 &  1\\ \end{array}\right] = \bigl [ \delta _{i+1,j} - \delta _{ij} \bigr ] \in \mathbb {R}^{m \times (n + 2)} . \end{aligned}$$

Due to zero boundary conditions, $\bar{u}_0 = 0 = \bar{u}_{n+1}$, we can drop the two boundary nodes and work with $u = (\bar{u}_1, \dots , \bar{u}_n)$, defined on the *n* inner nodes, obtaining the equation $\varepsilon = B u$ with the injective matrix

$$\begin{aligned} B = \left[ \begin{array}{ccc} 1 \\ -1 & \quad \ddots \\ & \quad \ddots & \quad 1 \\ & \quad & \quad -1 \end{array}\right] {{} = \bigl [ \delta _{ij} - \delta _{i,j+1} \bigr ]} \in \mathbb {R}^{m \times n} . \end{aligned}$$

Denoting by $h = {{\,\textrm{meas}\,}}(K_i) = L/m$ the common length of each element, we have $D = h I$, $W_e= c h I$, $W_s= \frac{h}{c} I$, and $W_{\varepsilon }= \frac{c}{h} I$. Reverting to the notation $W_e$ for $W_{\varepsilon }$, this gives

$$\begin{aligned} B^TW_eB = \frac{c}{h} \left[ \begin{array}{cccc} 2 & \quad -1 \\ -1 & \quad \ddots & \quad \ddots \\ & \quad \ddots & \quad \ddots & \quad -1 \\ & \quad & \quad -1 & \quad 2 \end{array}\right] {} = \frac{c}{h} \bigl [ 2 \delta _{ij} - \delta _{i+1,j} - \delta _{i,j+1} \bigr ] \in \mathbb {R}^{n \times n} . \end{aligned}$$

The inverse of this product has the explicit form

$$\begin{aligned} (B^TW_eB)^{-1} = \frac{h}{c} [\tilde{b}_{ij}]_{i,j=1}^n \quad \text {with}\quad \tilde{b}_{ij} = {\left\{ \begin{array}{ll} \frac{1}{m} j (m - i), &  i \ge j, \\ \frac{1}{m} i (m - j), &  i \le j, \end{array}\right. } \end{aligned}$$

and the Moore–Penrose pseudoinverse $(B^TW_eB)^{-1} B^TW_e$ evaluates to

$$\begin{aligned} B^+ = \frac{1}{m} \left[ \begin{array}{cccccc} n & \quad -1 & \quad \dots & \quad \dots & \quad -1 \\ n - 1 & \quad n - 1 & \quad -2 & \quad \dots & \quad -2 \\ \vdots & \quad \ddots & \quad \ddots & \quad \ddots & \quad \vdots \\ 2& \quad \dots & \quad 2 & \quad 1 - n & \quad 1 - n \\ 1 & \quad \dots & \quad \dots & \quad 1 & \quad -n \end{array}\right] {{} = \bigl [ b_{ji}^+ \bigr ]} \in \mathbb {R}^{n \times m} \hbox {with}, b_{ji}^+ = {\left\{ \begin{array}{ll} -\frac{1}{m} j, &  j < i, \\ \frac{1}{m} (m - j), &  j \ge i. \end{array}\right. } \end{aligned}$$

The associated complementary orthogonal projections finally read

$$\begin{aligned} P_{\text {im}B}= I - \frac{1}{m} E, \qquad P_{\text {im}B}^\perp = \frac{1}{m} E, \end{aligned}$$

where

$$\begin{aligned} E = \left[ \begin{array}{ccc} 1 & \quad \dots & \quad 1 \\ \vdots & \quad \ddots & \quad \vdots \\ 1 & \quad \dots & \quad 1 \end{array}\right] \in \mathbb {R}^{m \times m} . \end{aligned}$$

From this, we readily obtain $(B^T)^+ = (B^+)^T\in \mathbb {R}^{m \times n}$ and $P_{\ker B^T}= P_{\text {im}B}^\perp$ and hence $P_{\ker B^T}^\perp = P_{\text {im}B}$. Of course, these projections are also obtained from the observation that $(\text {im}B)^\perp = \ker B^T= {{\,\textrm{span}\,}}\{\textbf{1}\}$ where $\textbf{1}= (1, \dots , 1)^T\in \mathbb {R}^m$ and hence $E = \textbf{1}\textbf{1}^T$. The globally optimal discrete solutions of P1 finally satisfy

18 $$\begin{aligned} (\tilde{e}^*, \tilde{s}^*)&\in \mathop {\text {argmin}}\limits _{(\tilde{e}, \tilde{s}) \in \mathfrak {D}_{\textrm{sc}}^m} \frac{c}{m h} (\textbf{1}^T\tilde{e})^2 + \frac{h}{c} \Vert \tilde{s}- (\textbf{1}^T\tilde{s}/ m) \textbf{1}- (B^T)^+ q\Vert _2^2 \nonumber \\&= \mathop {\text {argmin}}\limits _{(\tilde{e}, \tilde{s}) \in \mathfrak {D}^m} \frac{c^2}{m} (\textbf{1}^T\tilde{e})^2 + \Vert \tilde{s}- (\textbf{1}^T\tilde{s}/ m) \textbf{1}- (B^T)^+ q\Vert _2^2 . \end{aligned}$$

This representation corresponds to (12); it formulates the global minimization as a “direct” finite problem. However, the number of choices in $\mathfrak {D}^m$ is $N^m$, which becomes astronomically large in practice.

We are now ready to show that our initialization is globally optimal for a uniform finite element discretization under symmetry of the force field and the data set.

#### Proposition 6

Suppose that

1. $$B = \bigl [\delta _{ij} - \delta _{i,j+1}\bigr ] \in \mathbb {R}^{m \times n};$$
2. $$W_e= c h^{-1} I = W_s^{-1}$$ with $$h > 0;$$
3. $q \in \mathbb {R}^n$ is symmetric in the sense that $q_{m-j} = q_j$ for $j = 1, \dots , n;$
4. $\mathfrak {D}$ is symmetric in the sense that $(\mathfrak {e}, \mathfrak {s}) \in \mathfrak {D}$ implies $-(\mathfrak {e}, \mathfrak {s}) \in \mathfrak {D}$.

Then, $(\tilde{e}_0, \tilde{s}_0)$ can be chosen to be globally optimal for problem P1.

#### Proof

Assumption (1) implies $(\text {im}B)^\perp = {{\,\textrm{span}\,}}\{\textbf{1}\} \subset \mathbb {R}^m_e$ and $\ker B^T= {{\,\textrm{span}\,}}\{\textbf{1}\} \subset \mathbb {R}^m_s$. Together with assumption (2), this gives $B^+ = \bigl [b_{ji}^+\bigr ]$ as stated above. Due to $b_{ji}^+ = -b_{m-j,m+1-i}^+$ and assumption (3), the vector $s_q = (B^T)^+ q \in \mathbb {R}^m$ then becomes antisymmetric in the sense that $(s_q)_{m+1-i} = -(s_q)_i$ for $i = 1, \dots , m$:

$$\begin{aligned} (s_q)_i + (s_q)_{m+1-i} = \sum _{j = 1}^n (b_{ji}^+ q_j + b_{j,m+1-i}^+ q_j) {\mathop {=}\limits ^{(3)}} \sum _{j = 1}^n (b_{ji}^+ q_j + b_{m-j,m+1-i}^+ q_j) = 0. \end{aligned}$$

By assumption (4), we may now choose $\tilde{s}_{0,m+1-i} = -\tilde{s}_{0i}$ when minimizing $|{\mathfrak {s}- s_{q,m+1-i}}|$ in initialization step (2), and similarly $\tilde{e}_{0,m+1-i} = -\tilde{e}_{0i}$. Then, $\tilde{s}_0 \in \mathbb {R}^m_s$ and $\tilde{e}_0 \in \mathbb {R}^m_e$ are also antisymmetric, which provides $\textbf{1}^T\tilde{s}_0 = \textbf{1}^T\tilde{e}_0 = 0$ and hence $(\tilde{e}_0, \tilde{s}_0) \in \text {im}B \times (\ker B^T)^\perp$. Thus, Proposition 5 applies.

#### Proposition 7

Suppose that P1 is a discretization of the DCQP (4) according to Section 2.1 with

1. a uniform mesh on $\Omega = (0, L);$
2. a symmetric force field $f \in L^2(\Omega )$ in the sense that $f(L - x) = f(x)$ for almost every $x \in \Omega ;$
3. a symmetric data set $\mathfrak {D}$.

Then, $(\tilde{e}_0, \tilde{s}_0)$ can be chosen to be globally optimal for problem P1.

#### Proof

A uniform mesh due to assumption (1) gives $B, W_e, W_s$ as required in Proposition 6, and for a symmetric field *f* due to assumption (2) it yields symmetric elemental, respectively, nodal forces $f \in \mathbb {R}^m$ and $q \in \mathbb {R}^n$. Thus, together with assumption (3), all prerequisites of Proposition 6 are satisfied.

A nonuniform discretization with respective element lengths $h_i = {{\,\textrm{meas}\,}}(K_i)$ gives $D = {{\,\textrm{Diag}\,}}[h_i]$, $W_e= c D$, $W_s= c^{-1} D$ and $W_{\varepsilon }= c D^{-1}$. Again, by reverting to the notation $W_e$ for $W_{\varepsilon }$, this gives

$$\begin{aligned} B^TW_e&= c \left[ \begin{array}{cccc} h_1^{-1} & \quad -h_2^{-1} \\ & \quad \ddots & \quad \ddots \\ & \quad & \quad h^{-1}_n & \quad -h^{-1}_m \end{array}\right] \in \mathbb {R}^{n \times m} , \\ B^TW_eB&= c \left[ \begin{array}{cccccc} h^{-1}_1 + h^{-1}_2 & \quad -h^{-1}_2 \\ -h_2^{-1} & \quad \ddots & \quad \ddots \\ & \quad \ddots & \quad \ddots & \quad -h^{-1}_n \\ & \quad & \quad -h^{-1}_n & \quad h^{-1}_n + h^{-1}_m \end{array}\right] \in \mathbb {R}^{n \times n} , \end{aligned}$$

but we no longer have simple analytic expressions for the matrix elements of $B^+$. Here one might employ a different variable scaling that obsoletes the weight matrices $W_e, W_s$ (i.e., the discretization-induced metrics) by scaling *B* instead, as follows. Using $V_e :=W_e^{1/2}$ and $V_s :=W_s^{1/2} = V^{-1}_e$, define

$$\begin{aligned} \varepsilon&:=V_e D e,&\tilde{\varepsilon }&:=V_e D \tilde{e},&\eta&:=V_e D \mu ,&\hat{B}&:=V_e B, \\ \sigma&:=V_s s,&\tilde{\sigma }&:=V_s \tilde{s},&q&:=C^Tf. \end{aligned}$$

The resulting DCQP with dual variables $\lambda$ (unscaled) and $\eta$ then takes the form

$$\begin{aligned} \min _{u,\varepsilon ,\sigma ,\tilde{\varepsilon },\tilde{\sigma } } \frac{1}{2} \Vert \varepsilon - \tilde{\varepsilon }\Vert _2^2 + \frac{1}{2} \Vert \sigma - \tilde{\sigma \Vert _2^2} \quad \text {s.t.}\quad \hat{B}^T\sigma - q&= 0, \\ \varepsilon - \hat{B} u&= 0, \end{aligned}$$

and the pseudoinverse is simply $\hat{B}^+ = (\hat{B}^T\hat{B})^{-1} \hat{B}^T$ with symmetric projection matrices

$$\begin{aligned} P_{\text {im}B}= \hat{B} \hat{B}^+ = (\hat{B} \hat{B}^+)^T= P_{\ker B^T}^\perp \quad \text {and}\quad P_{\text {im}B}^\perp = I - \hat{B} \hat{B}^+ = P_{\ker B^T}. \end{aligned}$$

This formulation is suitable for numerical computations: it involves only standard Euclidean norms and scalar products, and the additional scaling of *B* merely changes the numerical values while preserving the sparse structure.

### Data sets

In the numerical experiments, we use three types of data sets. For convergence studies with linear elasticity, we create artificial data sets with $N + 1$ evenly spaced exact data pairs $\mathfrak {e}^d = \mathfrak {s}^d \in [-1, 1]$:

$$\begin{aligned} \mathfrak {D}_N = \left\{ (-1, -1) + \tfrac{d}{N} (2, 2):d \in \mathcal {D}_N = \{0, \dots , N\}\right\} . \end{aligned}$$

For the second numerical experiment with a one-dimensional body, we use real measurement data that will be described in detail below. After removing 26 measurements, this data set contains only positive strain–stress pairs, and we complete it by adding all pairs with switched signs and moreover the origin. Finally, to compare our computations for the truss structure with the original approach in the third experiment, we copy the original data set by reading off the coordinates of each pair from [11, Fig. 2].

For each data set, we define the unit consistency factor *c* as the geometric mean of the positive quotients $\mathfrak {s}^d / \mathfrak {e}^d$:

$$\begin{aligned} c :=\exp \biggl ( \frac{1}{|{\mathcal {D}^+}|} \sum _{d \in \mathcal {D}^+} \ln \frac{\mathfrak {s}^d}{\mathfrak {e}^d} \biggr ) \quad \text {where}\quad \mathcal {D}^+ :=\{d \in \mathcal {D}:\mathfrak {e}^d \ne 0\}. \end{aligned}$$

The computations in [11] use the arithmetic mean (although it can be dominated by pairs with $\mathfrak {e}^d \approx 0$), and hence, we also employ the arithmetic mean when comparing results below,

$$\begin{aligned} c :=\frac{1}{|{\mathcal {D}^+}|} \sum _{d \in \mathcal {D}^+} \frac{\mathfrak {s}^d}{\mathfrak {e}^d}. \end{aligned}$$

We finally remark that a general requirement for DDCM computations is of course that the data used is “good enough” which, depending on the situation, may require further processing. For instance, when creating synthetic data with artificial noise, one may need to remove physically unreasonable pairs $(\mathfrak {e}^d, \mathfrak {s}^d)$ with opposite signs, $\mathfrak {e}^d \mathfrak {s}^d < 0$. When considering three-dimensional bodies, one may need to complete measured uni-axial or bi-axial data to obtain proper strain and stress tensors. We refrain from a further discussion as it is out of the scope of this work.

### Linear elasticity

Here we consider a unit-free academic test problem that has an analytic solution: a one-dimensional elastic beam of length *L* with a linear constitutive equation,

$$\begin{aligned} g(e, s) = s - \alpha e = 0, \end{aligned}$$

subjected to the sinusoidal force field

$$\begin{aligned} f(x) = f_0 \sin \frac{\pi x}{L}. \end{aligned}$$

One easily solves the differential equations $u' = e$, $s' = -f$ (in strong form) together with $s = \alpha e$ and boundary conditions $u(0) = 0 = u(L)$ to obtain the analytic solution

$$\begin{aligned} s^*(x)&= f_0 \frac{L}{\pi } \cos \frac{\pi x}{L} = \alpha e^*(x),&u^*(x)&= \frac{f_0}{\alpha } \frac{L^2}{\pi ^2} \sin \frac{\pi x}{L}. \end{aligned}$$

To keep the numerical experiments as simple as possible, we choose $L = \pi$ and $f_0 = \alpha = 1$, giving

$$\begin{aligned} f(x) = u^*(x)&= \sin x,&s^*(x) = e^*(x)&= \cos x. \end{aligned}$$

Recalling that the mesh is $0 = x_0< \dots < x_m = L$ with $K_i = [x_{i-1}, x_i]$ and $h_i = {{\,\textrm{meas}\,}}(K_i) = x_i - x_{i-1}$, the nodal shape functions can be written as

$$\begin{aligned} \phi _i(x) = \frac{x - x_{i-1}}{h_i} \chi _{K_i}(x) + \frac{x_{i+1} - x}{h_{i+1}} \chi _{K_{i+1}}(x), \quad i = 1, \dots , n. \end{aligned}$$

The nodal forces defined as $q = C^Tf$ become

19 $$\begin{aligned} q_i = \frac{1}{2} (h_i f_i + h_{i+1} f_{i+1}), \quad i = 1, \dots , n. \end{aligned}$$

Next, we consider continuous error measures for a triple $(u, e, s) \in \mathbb {R}^{n + 2 m}$ with associated functions $(u_h, e_h, s_h)$. The $L^2$ error of $u_h = \sum _{i = 1}^n u_i \phi _i$ evaluates to

$$\begin{aligned} \Vert {u_h - u^*}\Vert _{{K_i}}^2&= \frac{h_i}{3} (u_{i-1}^2 + u_{i-1} u_i + u_i^2) + 2 (u_i \cos x_i - u_{i-1} \cos x_{i-1}) \\&\quad - 2 \frac{u_i - u_{i-1}}{h_i} (\sin x_i - \sin x_{i-1}) + \frac{h_i}{2} - \frac{1}{4} (\sin 2 x_i - \sin 2 x_{i-1}). \end{aligned}$$

For $i = 1$ some terms vanish since $x_0 = 0$ and $u_0 = 0$, similarly for $i = m$ since $x_m = \pi$ and $u_m = 0$. The $L^2$ error of $e_h = \sum _{i = 1}^m e_i \chi _{K_i}$ is

$$\begin{aligned} \Vert {e_h - e^*}\Vert _{{K_i}}^2&= h_i e_i^2 - 2 e_i (\sin x_i - \sin x_{i-1}) + \frac{h_i}{2} + \frac{1}{4} (\sin 2 x_i - \sin 2 x_{i-1}). \end{aligned}$$

The same error formula (with $e_i$ replaced by $s_i$) is valid for $s_h = \sum _{i = 1}^m s_i \chi _{K_i}$.

As suitable discrete error measures, we use best $L^2$ approximations $(u_h^*, e_h^*, s_h^*)$ to $(u^*, e^*, s^*)$. The best $L^2$ approximation $e_h^*$ to $e^*$ has coefficients $e_i^* = h^{-1}_i (\sin x_i - \sin x_{i-1}) = h^{-1}_i \int _{K_i} e^*(x) \,dx$, and similarly for $s_h^*$. The best $L^2$ approximation $u_h^*$ to $u^*$ is obtained by setting the partial derivatives of $\Vert {u_h - u^*}\Vert _{{L^2(\Omega )}}^2$ with respect to the coefficients $u_i$ to zero. For $i = 1, \dots , m$ we have

$$\begin{aligned} \frac{\partial EMPTY}{\partial u_{i-1}} \Vert {u_h - u^*}\Vert _{{K_i}}^2&= \frac{2}{3} h_i u_{i-1} + \frac{1}{3} h_i u_i - 2 \cos x_{i-1} + \frac{2}{h_i} (\sin x_i - \sin x_{i-1}), \\ \frac{\partial EMPTY}{\partial u_i} \Vert {u_h - u^*}\Vert _{{K_i}}^2&= \frac{1}{3} h_i u_{i-1} + \frac{2}{3} h_i u_i + 2 \cos x_i - \frac{2}{h_i} (\sin x_i - \sin x_{i-1}). \end{aligned}$$

Since $u_0 = u_m = 0$, this gives for $i = 1, \dots , n$:

$$\begin{aligned} \frac{\partial EMPTY}{\partial u_i} \Vert {u_h - u^*}\Vert _{{L^2(\Omega )}}^2&= \frac{1}{3} h_i u_{i-1} + \frac{2}{3} h_i u_i + 2 \cos x_i - \frac{2}{h_i} (\sin x_i - \sin x_{i-1}) + {} \\&\quad \; \frac{2}{3} h_{i+1} u_i + \frac{1}{3} h_{i+1} u_{i+1} - 2 \cos x_i + \frac{2}{h_{i+1}} (\sin x_{i+1} - \sin x_i). \end{aligned}$$

We finally obtain the coefficients $u_i^*$ from the symmetric positive definite tridiagonal linear system

$$\begin{aligned} \left[ \begin{array}{cccc} 2 h_1 + 2 h_2 & \quad h_2 \\ h_2 & \quad \ddots & \quad \ddots \\ & \quad \ddots & \quad \ddots & \quad h_n \\ & \quad & \quad h_n & \quad 2 h_n + 2 h_m \end{array}\right] \left( \begin{array}{c} u_1^* \\ \vdots \\ \vdots \\ u_n^* \end{array}\right) = 6 \left( \begin{array}{c} e_1^* - e_2^* \\ \vdots \\ \vdots \\ e_n^* - e_m^*\\ \end{array}\right) . \end{aligned}$$

Given a numerical solution $(u, e, s) \in \mathbb {R}^{n + 2 m}$ of problem P1 with associated functions $(u_h, e_h, s_h)$, we will thus compute the continuous error norms

$$\begin{aligned} \Vert {u_h - u^*}\Vert _{{L^2(\Omega )}}, \qquad \Vert {e_h - e^*}\Vert _{{L^2(\Omega )}}, \qquad \Vert {s_h - s^*}\Vert _{{L^2(\Omega )}} \end{aligned}$$

(where $u^*, e^*, s^*$ refer to the analytic solutions in function space) and the discrete error norms

$$\begin{aligned} \Vert {u - u^*}\Vert _{{\bar{D}}} \quad \text {with}\quad \bar{D}_i :=\frac{1}{2} (h_i + h_{i+1}), \qquad \Vert {e - e^*}\Vert _{{W_e}}, \qquad \Vert {s - s^*}\Vert _{{W_s}}, \end{aligned}$$

(where $u^*, e^*, s^*$ refer to the coefficient vectors of the best $L^2$ approximations $u_h^*, e_h^*, s_h^*$). The scaling matrix $\bar{D}$ for *u* arises because node *i* is common to elements *i* and $i + 1$; this corresponds to (19). Moreover, since $c = 1$ for each exact data set $\mathfrak {D}_N$, we will have $W_e= c D = D$ and $W_s= c^{-1} D = D$.

Using the exact data sets $\mathfrak {D}_N$ for $N \in \{10^1, \dots , 10^6\}$ and uniform meshes on $[0, L] = [0, \pi ]$ with $m \in \{10^1, \dots , 10^5\}$ elements, the resulting discretized problems satisfy the assumptions of Proposition 7 in all cases, and our structure-specific initialization will always produce global minimizers. The ADM with this initialization gives the errors displayed in Table 1. The computed errors converge monotonically to zero when increasing both *m* and *N*. For the stress *s*, all errors are independent of *N* and converge monotonically when increasing *m*, whereas for *u* and *e* they converge nonmonotonically to a positive limit when increasing just one value and fixing the other. Essentially the same behavior occurs with a zero initialization of the ADM (not shown in the table), but all errors are strictly larger in this case as expected, except for the stress *s* whose errors are all identical to our results.

**Table 1:** Error norms for linearly elastic beam with *m* uniform finite elements and exact data sets $\mathfrak {D}_N$. Errors of $s_h$ and, respectively, *s* are identical for all values of *N*

*N*	$m=10^1$	$m=10^2$	$m=10^3$	$m=10^4$	$m=10^5$
			$$\Vert {u_h - u^*}\Vert _{{L^2(\Omega )}}$$		
$10^1$	2.083e-02	8.246e-03	1.341e-02	1.332e-02	1.329e-02
$10^2$	1.557e-02	4.351e-04	4.416e-04	4.458e-04	4.269e-04
$10^3$	1.550e-02	1.539e-04	4.504e-05	1.475e-05	1.367e-05
$10^4$	1.614e-02	1.655e-04	2.040e-06	7.372e-07	3.210e-07
$10^5$	1.610e-02	1.607e-04	9.127e-07	1.910e-07	NaN
$10^6$	1.610e-02	1.613e-04	1.678e-06	NaN	NaN
			$$\Vert {e_h - e^*}\Vert _{{L^2(\Omega )}}$$		
$10^1$	1.629e-01	9.748e-02	9.678e-02	9.677e-02	9.677e-02
$10^2$	1.139e-01	1.482e-02	1.013e-02	1.006e-02	1.006e-02
$10^3$	1.136e-01	1.141e-02	1.528e-03	1.018e-03	1.018e-03
$10^4$	1.136e-01	1.137e-02	1.141e-03	1.525e-04	1.028e-04
$10^5$	1.136e-01	1.137e-02	1.137e-03	1.141e-04	1.527e-05
$10^6$	1.136e-01	1.137e-02	1.137e-03	1.137e-04	1.141e-05
			$$\Vert {s_h - s^*}\Vert _{{L^2(\Omega )}}$$		
any	1.136e-01	1.137e-02	1.137e-03	1.137e-04	1.142e-05
			$$\Vert {u - u^*}\Vert _{{\bar{D}}}$$		
$10^1$	2.531e-02	8.339e-03	1.341e-02	1.332e-02	1.329e-02
$10^2$	1.502e-02	4.495e-04	4.418e-04	4.458e-04	4.269e-04
$10^3$	1.490e-02	1.473e-04	4.506e-05	1.475e-05	1.370e-05
$10^4$	1.558e-02	1.589e-04	1.992e-06	7.376e-07	9.593e-07
$10^5$	1.554e-02	1.540e-04	7.877e-07	1.922e-07	9.037e-07
$10^6$	1.554e-02	1.546e-04	1.613e-06	1.398e-08	9.034e-07
			$$\Vert {e - e^*}\Vert _{{W_e}}$$		
$10^1$	1.169e-01	9.681e-02	9.677e-02	9.677e-02	9.677e-02
$10^2$	1.017e-02	9.510e-03	1.006e-02	1.006e-02	1.006e-02
$10^3$	4.609e-03	9.464e-04	1.021e-03	1.018e-03	1.018e-03
$10^4$	5.220e-03	1.179e-04	1.026e-04	1.017e-04	1.022e-04
$10^5$	5.175e-03	5.201e-05	1.030e-05	1.032e-05	1.020e-05
$10^6$	5.177e-03	5.154e-05	1.174e-06	1.023e-06	1.021e-06
			$$\Vert {s - s^*}\Vert _{{W_s}}$$		
any	5.177e-03	5.154e-05	5.154e-07	5.154e-09	3.361e-10

The nonmonotonic convergence of variables *u* and *e* is not surprising since the DCQP involves discrete optimization variables. With *m* fixed, the solutions must converge to $e = s = (B^T)^+ q$ and $u = (B^TB)^{-1} q$ as $N \rightarrow \infty$. With *N* fixed and $m \rightarrow \infty$, it is not straightforward to compute the limits. The observation that stress errors are independent of *N* is explained by the symmetries of the force field and data. We have $s^*(L - x) = -s^*(x)$ for the optimal continuous solution and $(-\mathfrak {e}, -\mathfrak {s}) \in \mathfrak {D}$ if $(\mathfrak {e}, \mathfrak {s}) \in \mathfrak {D}$, hence also $\tilde{s}^*(L - x) = -\tilde{s}^*(x)$. For the discretization, we then get the data-independent stresses

$$\begin{aligned} (B^T)^+ q = s_q = s^* \in (\ker B^T)^\perp \quad \text {with}\quad \textbf{1}^Ts_q = \textbf{1}^Ts^* = 0. \end{aligned}$$

Because of the data symmetry, we also have

$$\begin{aligned} \textbf{1}^T\tilde{s}^* = \textbf{1}^T\tilde{e}^* = 0 \end{aligned}$$

with $\tilde{s}^* = \tilde{s}_0$ and $\tilde{e}^* = \tilde{e}_0$ (cf. Proposition 6), giving by (18) the globally optimal objective value that is actually computed numerically:

$$\begin{aligned} \frac{c}{m h} (\textbf{1}^T\tilde{e}^*)^2 + \frac{h}{c} \Vert \tilde{s}^* - (\textbf{1}^T\tilde{s}^* / m) \textbf{1}- (B^T)^+ q\Vert _2^2 = \frac{h}{c} \Vert \tilde{s}_0 - s_q\Vert _2^2. \end{aligned}$$

With our new initialization, we finally observe that the ADM terminates after the first iteration in all cases, reproducing iterate zero from the initialization without any changes. Moreover, the strain objective is zero up to roundoff errors. This confirms that our initialization already produces a global DCQP minimizer. For the case with $m = 10$ and $N = 10$, we have further confirmed global optimality by comparing optimal values for all $10^{10}$ combinations of the discrete variables $(\tilde{e}, \tilde{s})$ using (18). For all larger values of *m* and *N*, such a direct check is out of reach. However, Gurobi (version 11.0.3) confirmed global optimality of all results with $m = 10$ and $N \in \{10^1, \dots , 10^6\}$ using the MIQP formulation of [11] and our optimal solutions as initial estimates.

The largest computing times (wall clock, for $m = 10^5$ and $N = 10^6$) are 212.1s for one ADM iteration with our new initialization and 4106s for 20 ADM iterations with zero initialization using a single thread. For $m = 10$ and $N = 10^6$, the ADM needs 11ms with our initialization while Gurobi needs 200.2s using all eight threads, that is, roughly 1602s in total. This gives a factor well over $10^5$. With $m = 100$, Gurobi runs out of memory even for $N = 10$ with an objective at 24.3% of the initial value after 55:30 min using all eight threads (that is, 7:26 h total computing time), having explored almost 68 million nodes of the branch-and-bound tree and having already generated over 54 million further nodes.

### Nonlinear elasticity with real measurements

**Fig. 1:**
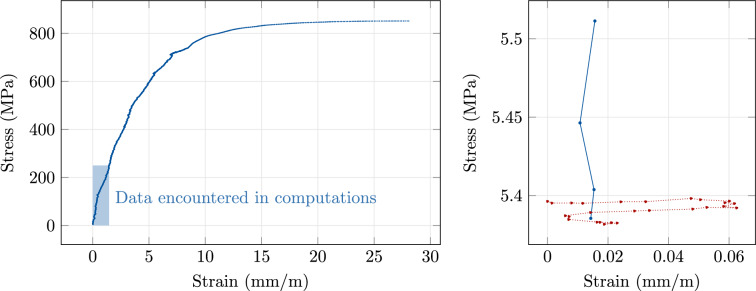
Left: measured data for 17-4 PH steel. Right: zoom into data pairs 1–26 (dotted, removed) and 27–30 (solid, kept)

In this section, we use a real data set consisting of 3775 data pairs for 17-4 PH stainless steel. The measurements were obtained at the Institute of Continuum Mechanics of Leibniz Universität Hannover by conducting a uni-axial tensile test according to the DIN EN ISO 6892-1 standard with a flat steel sample according to the DIN 50125-E standard. The data set is illustrated in Fig. 1. It covers the elastic and plastic regions up to the tensile strength; we have manually removed the first 26 data pairs, and we omit 150 further data pairs in the necking region until fracture. All data pairs that are actually encountered during our computations are located in the elastic region.

**Fig. 2:**
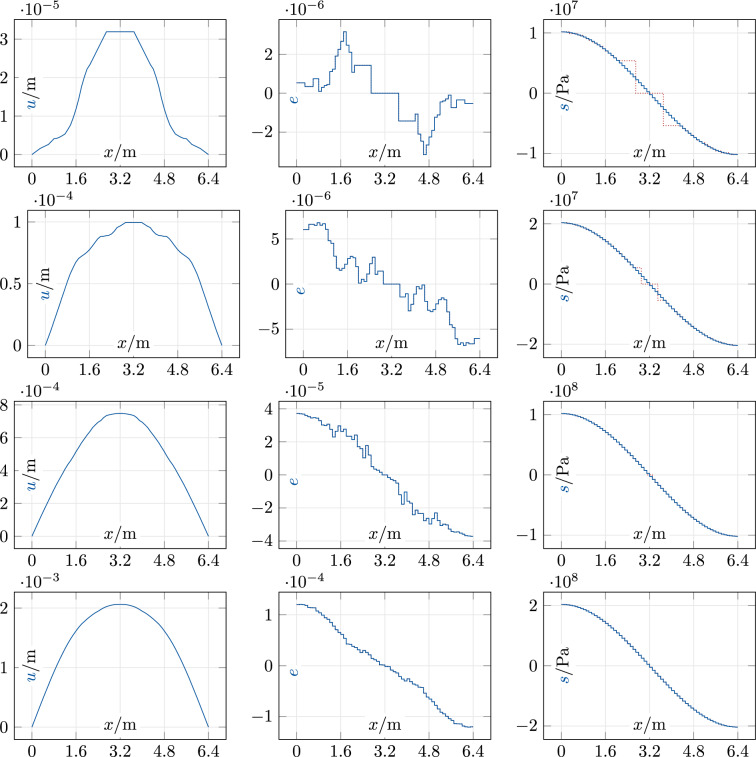
Steel bar: sinusoidal force, $f_0 \in \{5, 10, 50, 100\}$ MN/m (top to bottom). Left: *u*; middle: $e, \tilde{e}$ (solid blue, dotted red); right: $s, \tilde{s}$ (solid blue, dotted red)

We consider a steel bar of length $L = {6.4}\hbox {m}$ with a cross-sectional area $A = {1}\hbox {m}^{2}$, discretized into $m = 64$ elements of length 0.1m each, on which the sinusoidal force field with several values of $f_0$ acts. Solutions with our structure-specific ADM initialization are shown in Fig. 2. As in the linearly elastic case, the ADM terminates in all runs after the first iteration and reproduces iterate zero from the initialization, and the strain objective is zero up to roundoff errors. Here we have the same symmetries as before (hence globally optimal solutions) but real measured data, which confirms that exact data are entirely irrelevant for the global optimality of our initialization. In fact, the irregularity of the measured data does not affect the optimal stress profiles (right side in Fig. 2) but is clearly visible in the strains and displacements (middle and left). Naturally, the cases with small force maximum $f_0$ produce solutions with small displacements, strains, and stresses, resulting in large irregularity and relatively large jumps of the discrete fields $(\tilde{e}, \tilde{s})$ since the relative density of relevant data points is small. Accordingly, the difference between continuous and discrete stresses *s* and $\tilde{s}$ is relatively large close to zero and very small otherwise. As mentioned and clearly visible, the differences between continuous and discrete strains *e* and $\tilde{e}$ are zero up to roundoff errors.

**Fig. 3:**
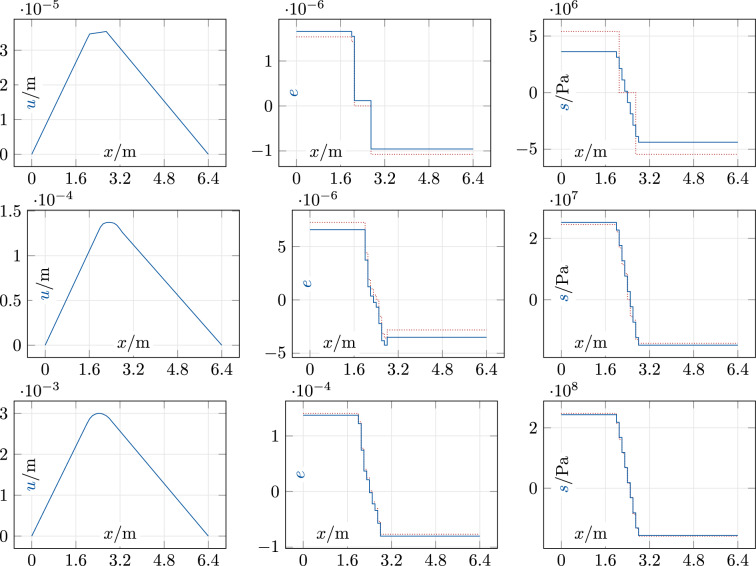
Steel bar: block at [2.0, 2.8] m, $f_0 \in \{10, 50, 500\}$ MN/m (top to bottom). Left: *u*; middle: $e, \tilde{e}$ (solid blue, dotted red); right: $s, \tilde{s}$ (solid blue, dotted red)

**Fig. 4:**
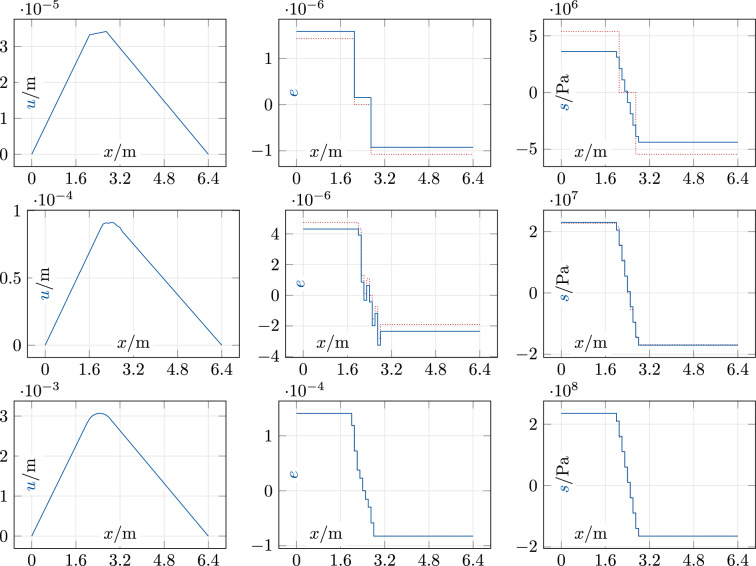
Steel bar, zero initialization: block force, $f_0 \in \{10, 50, 500\}$ MN/m (top to bottom). Left: *u*; middle: $e, \tilde{e}$ (solid blue, dotted red); right: $s, \tilde{s}$ (solid blue, dotted red)

Solutions for a rectangular “block” of length 0.8m centered at 2.4m, which creates a nonsmooth and asymmetric force field of the (unit-free) form $f(x) = f_0 \chi _{[2.0,2.8]}(x)$, are shown in Fig. 3. Here, the data irregularity around the origin is again apparent. For the first two load cases, the ADM with our new initialization converges in three iterations where all 64 data pairs change at the first iteration and one more pair changes at the second. For the third load case with $f_0 = {500}\hbox {MN/m}$, the ADM needs eight iterations with 116 changes of data pairs in total. In summary, the ADM with our structure-specific initialization converges quickly in all cases. Considering the large data set, it produces unexpectedly few changes of the discrete variables. Our initialization is clearly non-optimal on these asymmetric problems, but the resulting solutions are physically plausible. However, solutions of the zero-initialized ADM (illustrated in Fig. 4) have better objective values on the rectangular force profile: just slightly smaller for $f_0 = {10}\hbox {MN/m}$ and drastically smaller for $f_0 = {500}\hbox {MN/m}$; see Table 2. Although this is slightly disappointing, one can of course not expect that any non-optimal initialization is consistently better on all problems in an ADM setting.

The largest computing times are 4ms with the new initialization and 8ms with zero initialization. We are unable to compute a global minimizer with Gurobi for any of the seven problem instances.

### Truss structure

**Fig. 5:**
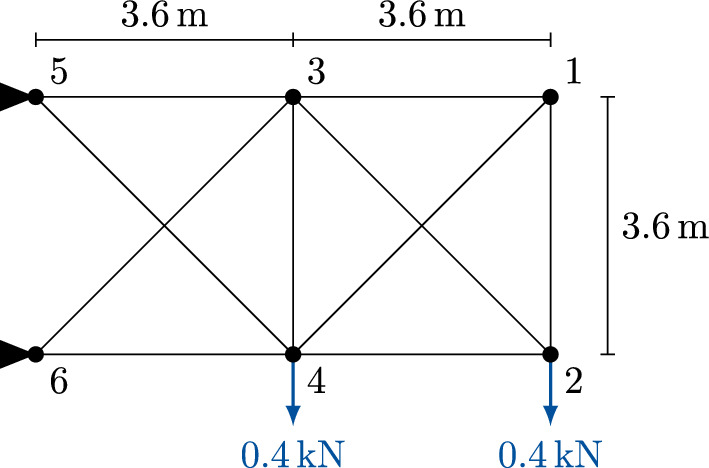
10-bar truss structure taken from [11]

Here we consider the two-dimensional truss structure depicted in Fig. 5, which was used as a test problem for the MIQP reformulation of the DCQP by Kanno [11]. The structure connects six nodes with $m = 10$ truss elements. Nodes 5 and 6 are fixed, resulting in $n = 8$ degrees of freedom for the coordinates of the remaining four nodes. The cross-sectional area of each truss element is 0.002${\hbox {m}^{2}}$, and downward forces of $f_0 = {0.4}\hbox {kN}$ are applied at nodes 2 and 4. The truss structure is assumed to experience one-dimensional mechanical deformations without any bending of the elements. In the mathematical model, the matrix *D* simply contains the lengths of all truss elements (or *members*). The matrix *B* is constructed as follows. Let $\mathcal {N}$ denote the set of nodes, $\mathcal {E}$ the set of edges (members), and $E \in \mathbb {R}^{m \times |{\mathcal {N}}|}$ the *arc-node incidence matrix* of the truss graph $G = (\mathcal {N}, \mathcal {E})$, where $E_{ij} = -1$ if edge *i* starts in node *j*, $E_{ik} = +1$ if edge *i* ends in node *k*, and $E_{i\ell } = 0$ otherwise. Replace each zero entry with $0 \in \mathbb {R}^2$ and each $\pm 1$ entry with the direction vector of its edge, $\pm (x_k - x_j) / \Vert {\in }\Vert _{{x_k - x_j}}\mathbb {R}^2$. Delete all columns associated with fixed node coordinates to obtain $B \in \mathbb {R}^{m \times n}$.

For his numerical experiments, Kanno considered gradually increasing forces $\lambda f_0$ with amplification factor $\lambda \in \{0, 1, \dots , 11\}$. The ADM for $\lambda = 0$ was started with $s_q$ initialized to zero, whereas for all values $\lambda > 0$ the ADM was warm-started with the solution obtained for $\lambda - 1$.

The (artificial) data set consists of 300 pairs that are not symmetric with respect to the origin. As already mentioned, we replicated the original data set by extracting the coordinates of each pair from [11, Fig. 2]. We dropped two pairs with opposite signs, $\mathfrak {e}^d \mathfrak {s}^d < 0$. The resulting compatibility factor is $c \approx {1.747}\hbox {GPa}$. For all 300 pairs, the value becomes 1.654GPa while [11] reports a value of 1.622GPa.

**Table 2:** Comparisons for real data: sinusoidal force top, block force bottom

	Structure-specific initialization					Zero initialization				
$f_0$	obj-*e*	obj-*s*	obj	iter	mod	obj-*e*	obj-*s*	obj	iter	mod
5	0.000	19.712	19.712	1	0	0.000	22.766	22.766	5	76
10	0.000	9.567	9.567	1	0	0.000	71.187	71.187	4	86
50	0.000	4.632	4.632	1	0	0.000	36.211	36.211	11	284
100	0.000	0.242	0.242	1	0	0.000	86.710	86.710	17	452
10	0.629	46.725	47.354	3	60	1.036	46.259	47.294	3	81
50	20.74	16.19	36.93	2	63	8.424	1.641	10.065	5	163
500	493.84	239.77	733.61	8	116	0.574	6.969	7.543	15	708

The results are given in Table 3 where we compare the two individual objective terms, the total objective, and the number of iterations for ADM runs with our structure-specific initialization versus zero-initialized ADM ($\lambda = 0$), respectively, warm-started ADM runs ($\lambda > 0$). For reference, the global minimum obtained with Gurobi (again initialized with our ADM solution) is also given.

**Table 3:** Computational results for the 10-bar truss experiment

	Structure-specific initialization				Kanno’s initialization [11]				Gurobi
$\lambda$	obj-*e*	obj-*s*	obj	iter	obj-*e*	obj-*s*	obj	iter	obj
0	0.187	485.473	485.66	1	0.187	485.473	485.66	1	280.43
1	15.6016	47.8511	63.4527	2	28.5028	197	225.503	2	39.62
2	16.1438	50.1955	66.3393	1	0.53029	244.616	245.146	3	47.43
3	7.08256	6.60861	13.6912	3	14.9918	47.6	62.5918	3	13.01
4	56.4908	34.8507	91.3415	2	18.387	235.56	253.95	5	36.77
5	14.876	30.923	45.799	1	22.7738	224.011	246.785	3	15.82
6	39.5887	27.9889	67.5777	2	9.94678	164.07	174.017	3	20.66
7	74.5353	54.0956	128.631	2	102.103	185.72	287.823	3	79.64
8	31.4766	110.863	142.339	2	72.06	291.36	363.42	7	74.17
9	43.9127	73.8742	117.787	1	23.9522	852.082	876.035	4	50.46
10	5.80513	5.82575	11.6309	3	146.979	489.962	636.941	5	5.92
11	20.0655	27.4347	47.500	2	3.78673	1028.899	1032.686	4	9.05

In the unloaded case, both ADM approaches produce identical results: our new initialization becomes a zero-initialization since $q = 0$ implies $s_q = 0$. In all other cases, our initialization gives consistently (and often substantially) better results than the warm start in terms of the total objective (obj). Moreover, our stress objective (obj-s) is also consistently smaller, whereas the strain objective (obj-e) is larger for $\lambda \in \{2, 4, 6, 9\}$. Finally, our number of iterations is also consistently equal or smaller. In conclusion, the structure-specific initialization outperforms the warm-start in all cases, although there is no symmetry whatsoever and the initialization is again clearly non-optimal.

In this experiment, all computing times of the two ADM approaches are below 0.5ms using one thread while Gurobi needs between 4.91s and 13.40s using eight threads (one per physical core) to reach and prove global optimality.

**Fig. 6:**
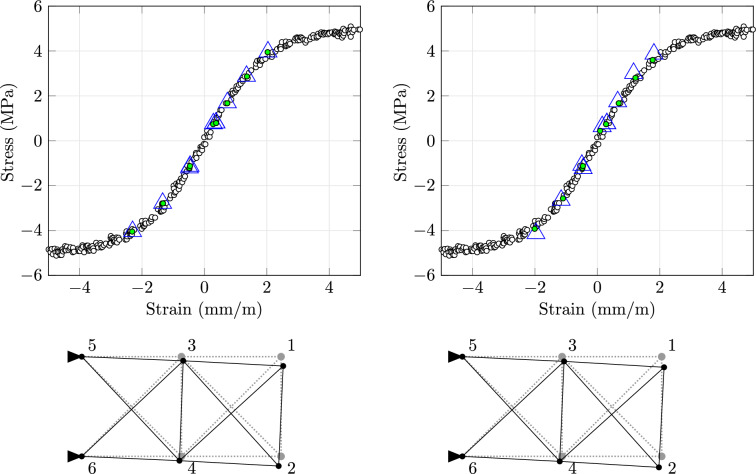
ADM solutions for $\lambda = 10.0$ with our structure-specific initialization (left) and with Kanno’s initialization (right) as in [11]. Top: Triangles represent the stress and strain of each member, while filled circles indicate the closest data points. Bottom: resulting 10-bar truss structures in undeformed state (dotted) and deformed state (solid), with all displacements $u_i$ scaled up by a factor of 10

A visualization of the solutions is provided in Fig. 6. We observe that our strains and stresses of the ten truss elements (the centers of blue triangles in the left upper part) lie closer to the data set and closer to their associated data pairs (highlighted circles) than the corresponding values in the right upper part. Each difference between a triangle center and its associated circle corresponds to an individual contribution to each objective, and the large differences in the vertical direction of the upper right part clearly show the dominating stress objective of the warm-started ADM. With our approach, the triangles are visibly better centered on their associated data points, which reflects the much smaller objective values (roughly $6 + 6 = 12$) in comparison with the warm-started values (roughly $147 + 490 = 637$). The lower part of Fig. 6 shows the corresponding displacements of the entire structure, exaggerated by a factor of ten to make them better visible. Here, marginally larger displacements appear to be discernible on the right side, but it is impossible to tell which side shows the more accurate solution.

## Conclusion

In this work, we investigated the mathematical structure and numerical solution of data-driven elasticity problems that are defined on a closed interval of the real line and discretized by means of the finite element method. For such an end, we provided a complete structural analysis of the underlying DCQP and of an associated penalty formulation. Even when globally solvable, the solution of the DCQP by enumeration (i.e., comparing all possible objective values) is not feasible. Moreover, a standard mixed-integer QP reformulation can be rigorously solved to global optimality by branch-and-bound methods as implemented in Gurobi, but only for very small problem instances, that is, coarse discretizations. Following the mainstream, we therefore solved the DCQP by an ADM, which is known to work well for discrete–continuous problems. Nevertheless, such a method will not converge, in general, to a local or global minimizer even when each subproblem is solved to global optimality. Based on the formal structural analysis, another novel contribution of the current work is a new structure-specific initialization for the ADM that in certain symmetric cases yields *provably* global minimizers and in several other cases produced better solutions than cold or random initializations followed in the literature.

Even when this work represents a solid step toward a better understanding of the DCQPs that arise in the context of DDCM, there are still many open questions to be addressed in the future. The most urgent ones are perhaps those concerning the extension of the proposed analysis to: *i*) data-driven elasticity problems defined on closed subsets of the two- and three-dimensional ambient space and *ii*) primal and dual fields belonging to infinite-dimensional function spaces. Ultimately, the structural insights should be utilized to develop improved rigorous solution algorithms.

## Data Availability

Data will be made available on request.
